# Structural language profiles in children with autism spectrum disorder and developmental language disorder: a systematic review

**DOI:** 10.3389/fpsyg.2026.1920573

**Published:** 2026-09-03

**Authors:** Irine Doghadze, Tamar Gagoshidze

**Affiliations:** Faculty of Psychology and Educational Science, Ivane Javakhishvili Tbilisi State University, Tbilisi, Georgia

**Keywords:** autism spectrum disorder, developmental language disorder, language phenotype, morphosyntax, phonology, semantics, structural language, systematic review

## Abstract

**Background:**

Autism Spectrum Disorder (ASD) and Developmental Language Disorder (DLD) are both associated with language difficulties, yet the extent to which they share common structural language profiles remains unclear. Previous research has reported both overlap and divergence across phonological, morphosyntactic, semantic, and narrative domains, creating challenges for differential diagnosis and intervention planning.

**Objective:**

This systematic review aimed to synthesize empirical evidence comparing structural language abilities in children with ASD and DLD and to identify areas of convergence and divergence across language domains.

**Methods:**

A systematic literature search was conducted in PubMed, ERIC, PsycINFO, Google Scholar, and Semantic Scholar following PRISMA guidelines. Studies published in English between 2013 and 2025 were eligible if they included children aged approximately 3–18 years with ASD and DLD and examined structural language domains, including phonology, morphology, syntax, semantics, or narrative measures. 44 studies met the inclusion criteria and were synthesized using a narrative approach due to methodological heterogeneity.

**Results:**

The included studies suggested overlapping performance between children with ASD accompanied by language impairment (ASD-LI) and children with DLD across receptive and expressive language, morphosyntax, sentence repetition, nonword repetition, and complex syntactic processing. Overlapping performance was particularly evident in grammatical morphology, tense and aspect marking, clitic production, and comprehension of syntactically demanding structures. However, important differences emerged. Children with DLD generally exhibited more consistent and pervasive structural language deficits, whereas language performance in ASD was characterized by greater heterogeneity. Difficulties in ASD were more frequently influenced by pragmatic, attentional, cognitive, and processing-related factors, and atypical error patterns were observed in several linguistic domains.

**Conclusion:**

The available evidence suggests a partially overlapping structural language phenotype between children with ASD and co-occurring language impairment (ASD-LI) and those with DLD, while also highlighting important disorder-specific characteristics. Across several structural language domains, the included studies frequently reported similar performance on standardized language measures. The findings further suggest that similar behavioral performance may reflect distinct underlying linguistic and cognitive mechanisms, resulting in different patterns of language impairment. Overall, the evidence suggests that ASD-LI and DLD share several structural language characteristics but do not represent a single, unified language disorder. These findings have important implications for differential diagnosis, language assessment, and the development of targeted intervention approaches for children with ASD and DLD.

## Introduction

Language is central to communication and is commonly conceptualized in terms of three core components: form, content, and use (Andreou et al., 2025). Language form, which includes phonology, morphology, and syntax, together with language content (semantics), constitutes structural language, whereas language use is typically examined within the domain of pragmatics (Baird and Norbury, 2016; Reindal et al., 2021). To provide a more comprehensive description of language functioning, language is often examined across five interconnected domains: phonology, morphology, syntax, semantics, and pragmatics (Reetzke et al., 2015). Within this framework, structural language generally encompasses phonology, morphology, syntax, and semantics (Reetzke et al., 2015; Vassiliu et al., 2022).

Although dividing language into discrete linguistic levels is somewhat artificial, this approach remains highly valuable for analyzing and assessing language abilities, particularly in atypical populations (Matthews et al., 2018). Each domain captures distinct but interrelated aspects of linguistic competence. Phonology refers to the sound structure of language, including phonemes—the smallest units of sound that distinguish meaning—as well as prosodic features such as intonation (Stemberger and Bernhardt, 2023; Barokova and Tager-Flusberg, 2020). Morphology concerns the internal structure of words and focuses on morphemes, the smallest meaningful units of language, and the rules governing word formation (Barokova and Tager-Flusberg, 2020; James et al., 2021). Syntax refers to sentence structure and the rules that govern word order, enabling speakers to construct and interpret meaningful sentences (Fromkin et al., 2017). Semantics focuses on meaning at the level of words, phrases, and sentences and is supported by the individual’s mental lexicon (Sun and Poeppel, 2023).

Language development is a complex, multidimensional process, and disruptions in this process are central to several neurodevelopmental conditions, most notably Autism Spectrum Disorder (ASD) and Developmental Language Disorder (DLD). Although both conditions are associated with language-related difficulties, the nature, profile, and underlying mechanisms of these impairments differ substantially, posing ongoing challenges for differential diagnosis, assessment, and intervention.

ASD is primarily characterized by persistent difficulties in social communication and interaction, alongside restricted and repetitive patterns of behavior (American Psychiatric Association, 2013). Language abilities in children with ASD are highly heterogeneous, ranging from minimal verbal output to age-appropriate structural language accompanied by marked pragmatic impairments (Tager-Flusberg et al., 2005; Tager-Flusberg, 2016). This variability is also evident in broader cognitive profiles, with substantial differences across individuals in both language abilities and cognitive strengths and challenges (Girolamo et al., 2024; Silleresi, 2023). Such heterogeneity extends to structural language skills, with some children demonstrating intact abilities and others showing significant impairments (Baird et al., 2006). Although structural language deficits are not included as a core diagnostic criterion in the DSM-5, a substantial proportion of children with ASD—approximately 65%—present with co-occurring language impairments (ASD-LI) and may receive an additional diagnosis of a language disorder (Schaeffer et al., 2023).

Language Disorder, as defined in the Diagnostic and Statistical Manual of Mental Disorders (5th ed.; DSM-5), refers to persistent difficulties in the acquisition and use of language across modalities due to deficits in comprehension or production, which significantly interfere with functioning and cannot be explained by other conditions (American Psychiatric Association, 2013). Although the term Developmental Language Disorder (DLD) is not formally included in the DSM-5, it has become the preferred term following the CATALISE international consensus (Bishop et al., 2017). CATALISE recommended replacing the term Specific Language Impairment (SLI) with DLD to describe persistent language difficulties that have a functional impact and are not associated with known biomedical conditions, such as intellectual disability, hearing loss, or neurological disorders. Importantly, autism spectrum disorder is not included within DLD under the CATALISE framework, although language impairment may co-occur with ASD.

Language difficulties are a defining characteristic of DLD and are also common in individuals with ASD (Marini et al., 2020; Roberts et al., 2004; Tager-Flusberg and Joseph, 2003; Georgiou and Spanoudis, 2021; Cohen and Volkmar, 1997; Riches et al., 2010; Williams et al., 2008). While some children with ASD exhibit relatively intact language abilities, others present with significant structural language impairments that closely resemble those observed in DLD.

Accordingly, a growing body of research has examined similarities between children with ASD and those with DLD across multiple levels of language development (Andreou and Aslanoglou, 2022; Leyfer et al., 2008; Ramírez-Santana et al., 2019; Tuller et al., 2017; Durrleman and Delage, 2016; Meir and Novogrodsky, 2020; Wittke et al., 2017). These overlaps have led to the proposal of a shared or overlapping language phenotype, suggesting that ASD and DLD may represent related conditions or different manifestations of partially shared underlying mechanisms (Bishop, 2010a,b; Kjelgaard and Tager-Flusberg, 2001; Tager-Flusberg and Joseph, 2003).

Particular attention has been given to children with ASD who present with additional language impairment (ASD-LI) despite typical cognitive abilities, as they often display language profiles similar to those observed in DLD (Tager-Flusberg, 2016; Durrleman and Delage, 2016). This similarity may contribute to diagnostic uncertainty, especially when assessment relies primarily on standardized language measures (Leyfer et al., 2008).

Empirical findings further support this overlap. Elevated levels of autistic-like traits and social-communication difficulties have been reported in some children with DLD, while a substantial proportion of children with ASD meet criteria for language impairment (Kjelgaard and Tager-Flusberg, 2001; Bishop, 2010a,b). Findings based on the Children’s Communication Checklist–2 (CCC-2) suggest considerable overlap between ASD and DLD communication profiles, with some children exhibiting intermediate patterns that blur traditional diagnostic boundaries (Bishop and Norbury, 2002; Bishop, 2003). Additionally, a subgroup of children with ASD demonstrates grammatical impairments comparable to those observed in DLD (Kjelgaard and Tager-Flusberg, 2001; Terzi et al., 2014; Durrleman et al., 2017). Together, these findings suggest partially overlapping phenotypic and potentially genetic mechanisms underlying language impairment across ASD and DLD (Bishop, 2010a,b; Tomblin et al., 1997).

DLD is primarily associated with structural language deficits, particularly in morphosyntax and phonology (Leonard, 1998, 2014; Bishop, 2010a,b), including difficulties with tense marking, agreement, wh-questions, and relative clauses (Rice et al., 1995; Wetherell et al., 2007; Jakubowicz, 2011), although broader cognitive and pragmatic difficulties have also been reported (Henry et al., 2012). Some studies report similar morphosyntactic and phonological deficits in both ASD and DLD (Durrleman and Delage, 2016; Tager-Flusberg, 2006, 2016), supporting the hypothesis of partial overlap between the disorders (Bishop, 2010a,b; Roberts et al., 2004).

However, accumulating evidence also points to important qualitative differences between ASD and DLD. ASD is more consistently associated with pragmatic impairments, atypical error patterns, and distinct cognitive profiles (Modyanova et al., 2017; Sukenik and Friedmann, 2018; Schaeffer, 2018), and current evidence does not support a clear shared familial etiology with DLD (Lindgren et al., 2009; Williams et al., 2008).

Consistent with this view, several studies have reported differences in the language profiles of children with ASD and DLD (Schaeffer, 2018; Williams et al., 2008; Whitehouse et al., 2008; Bishop, 2010a,b). For example, children with ASD have been shown to outperform those with DLD on nonword repetition tasks (Durrleman and Delage, 2016; Riches et al., 2011) and on certain lexical tasks involving word associations and lexical knowledge (Loucas et al., 2013; Manolitsi and Botting, 2011). Differences are also evident in sentence-level processing. Children with ASD differ from those with DLD in morphosyntactic error patterns and sentence processing strategies (Sukenik and Friedmann, 2018). In wh-question tasks, children with ASD tend to produce more pragmatically inappropriate responses, whereas children with DLD more commonly exhibit morphosyntactic errors, although both groups show difficulty with syntactically complex structures (Tuller et al., 2011).

From a developmental perspective, language profiles in ASD and DLD may converge in early childhood but diverge over time. Preschool children in both groups often display similar language difficulties; however, as development progresses, children with DLD tend to show more persistent structural language deficits, whereas children with ASD often exhibit increasing divergence in pragmatic and social-communicative functioning.

A systematic review focusing specifically on structural language is therefore needed. Although language impairments are common in both ASD and DLD, findings remain fragmented and often inconsistent across studies. Structural language domains - including phonology, morphology, syntax, and semantics - have frequently been investigated separately, using diverse assessment methods and heterogeneous participant groups. As a result, it remains unclear which language difficulties reflect shared characteristics across the two disorders and which represent distinct disorder-specific profiles. Synthesizing the available evidence may help clarify patterns of structural language impairment in ASD and DLD, improve differential diagnosis, and inform future research and intervention efforts.

### Structural language in ASD

Children with ASD exhibit highly variable language development trajectories, including regression, plateau, gradual improvement, and accelerated growth (Anderson et al., 2007; Kjelgaard and Tager-Flusberg, 2001). Language development is often delayed and heterogeneous, with impairments reported across lexical, syntactic, and morphological domains (Eigsti et al., 2011; Landa, 2007; Rapin and Dunn, 2003). Children with ASD frequently produce less complex syntax, rely on restricted grammatical structures, and show difficulties in grammatical judgment tasks (Eigsti et al., 2007; Eigsti and Bennetto, 2009; Rapin and Allen, 1983). Although phonological abilities are often less affected than other language domains, deficits have been reported in some subgroups of children with ASD (Bartak et al., 1975).

A defining feature of ASD is the marked heterogeneity in language abilities. Tager-Flusberg and Joseph (2003) identified two language subtypes among verbal children with ASD: one group with age-appropriate language abilities and another with significant language impairment. More recent research has suggested a broader range of language profiles. Vogindroukas et al. (2022) described four profiles, including ASD with primarily pragmatic difficulties, ASD with co-occurring DLD or other developmental disorders, ASD with intellectual disability, and ASD with social communication difficulties. Similarly, Silleresi (2023) described three commonly observed profiles: ASD with normal language (ASD-LN) and cognition, ASD with both language and intellectual impairment, and ASD with language impairment without intellectual disability (ASD-LI).

Standardized language assessments provide only a partial representation of linguistic competence in ASD, as testing contexts may not fully capture naturalistic communication and subtle language differences (Wiig et al., 2013). Individuals who perform within the normal range on standardized measures may nevertheless exhibit atypical performance on psycholinguistic tasks and in spontaneous language production (Eigsti and Bennetto, 2009; Kelley et al., 2006; Wittke et al., 2017). Qualitative and experimental studies have identified differences in lexical extension (Tek et al., 2008), pronominal reference (Zane et al., 2021), and semantic–pragmatic integration, even when morphosyntactic abilities appear relatively intact. Furthermore, online processing studies have revealed atypical neural and real-time responses to semantic and anaphoric information (Nagano et al., 2020). Consistent with these findings, Zane and Grossman (2024) demonstrated that ASD adolescents with average standardized language scores exhibit subtle but significant differences on more complex structural language tasks, suggesting qualitatively distinct language profiles that may not be captured by standardized assessments alone.

Children with ASD show heterogeneous phonological development, with some individuals demonstrating delays relative to typically developing peers (Schaeffer et al., 2023; Wolk et al., 2016). Despite these delays, the order of phoneme acquisition and the types of phonological errors produced are generally similar to those observed in typical development (Wetherby et al., 2004). Performance on nonword repetition tasks is often within the typical range, particularly among children with ASD who do not have co-occurring language impairment, although difficulties have been reported in some subgroups (Williams et al., 2013). Beyond phonological processing, lexical representations may develop atypically, with reduced retention of lexical and semantic information over time (Fletcher et al., 2020; Norbury et al., 2010). Phonological awareness has been identified as an important predictor of decoding skills in ASD and, in some studies, has shown stronger associations with reading outcomes than in typically developing peers (Dynia et al., 2017). Nevertheless, weaker performance on phonological awareness tasks has also been reported, highlighting considerable variability within the ASD population (Dynia et al., 2014; Hudson et al., 2017).

Morphological and syntactic development in ASD is highly heterogeneous, with a substantial subgroup exhibiting significant morphosyntactic difficulties. Children with ASD may show limited use of function words, including articles and pronouns, as well as difficulties with tense marking and grammatical rule generalization (Kelley et al., 2006; Vogindroukas, 2020). Cross-linguistic evidence indicates challenges in inflectional morphology, particularly in languages such as Greek (Terzi et al., 2014), and in aspect marking, where production may be impaired despite relatively preserved comprehension (Su and Naigles, 2023).

Many children with ASD produce shorter and less syntactically complex utterances and experience difficulties with complex grammatical constructions (Brynskov et al., 2017; Durrleman and Delage, 2016; Oi, 2008; Riches et al., 2010; Terzi et al., 2014; Tuller et al., 2017). Difficulties have been reported in passives, relative clauses, pronominal clitics, and wh-questions (Durrleman et al., 2017; Sukenik and Friedmann, 2018; Tuller et al., 2017; Prévost et al., 2018). These challenges have often been linked to the computational demands associated with syntactic movement, particularly in object relative clauses and object wh-questions (Friedmann and Haddad-Hanna, 2014). Although developmental trajectories may broadly parallel those of typically developing children, syntactic development in ASD is often delayed and appears to be influenced more strongly by nonverbal abilities than by chronological age (Durrleman et al., 2016).

Findings on binding are mixed. Children with ASD may show impairments in reflexive binding while demonstrating relatively intact interpretation of personal pronouns (Perovic et al., 2013), although cross-linguistic differences complicate interpretation (Terzi et al., 2014). Pronoun use is often atypical, including pronoun reversal and ambiguous reference, phenomena that are generally interpreted as reflecting pragmatic and discourse-level difficulties rather than core structural language deficits (Naigles et al., 2016; Evans and Demuth, 2012; Arnold et al., 2009; Norbury and Bishop, 2003; Novogrodsky and Edelson, 2016).

Lexical-semantic abilities in ASD are highly heterogeneous (Kjelgaard and Tager-Flusberg, 2001; de Marchena et al., 2011; Arunachalam and Luyster, 2016; Kwok et al., 2015). While performance on single-word vocabulary tasks is often relatively preserved, particularly among individuals without co-occurring language impairment (Luyster and Lord, 2009; Norbury et al., 2010), difficulties have also been reported in vocabulary breadth and depth, semantic organization, and the quality of lexical representations (McGregor et al., 2012; Norbury, 2005). Children with ASD may experience difficulties with abstract meanings, multiple word meanings, and deeper semantic processing (Rapin and Dunn, 2003; Horvath et al., 2018). Additional features include the use of neologisms, idiosyncratic expressions, and atypical word use (Eigsti et al., 2007), as well as reduced use of certain word classes such as numerals and pronouns (Liu et al., 2025).

Narrative tasks have frequently been used to assess structural language abilities in ASD because they require the integration of vocabulary and morphosyntactic knowledge in connected speech. Studies have reported reduced lexical diversity, shorter mean length of utterance, and lower syntactic complexity in the narratives of children with ASD compared with typically developing peers (Norbury and Bishop, 2003; Norbury et al., 2014). A large body of research has also documented difficulties in narrative production affecting both macrostructure and microstructure (Baixauli et al., 2016; Banney et al., 2015). Children with ASD often produce less coherent narratives, include fewer story grammar elements, and show reduced use of mental-state language and causal links within stories (Bang et al., 2012; Brown et al., 2013; Losh and Capps, 2003; Norbury et al., 2014). These difficulties have been reported across a wide range of ability levels and are frequently accompanied by impairments in referential cohesion and narrative content (Tager-Flusberg, 1995; Capps et al., 2000; Norbury and Bishop, 2003; Suh et al., 2014).

Importantly, narrative performance reflects not only structural language abilities but also discourse organization, pragmatic competence, and social-cognitive skills. Consequently, measures such as lexical diversity, mean length of utterance, grammatical accuracy, and syntactic complexity are often interpreted as indicators of structural language, whereas coherence, story grammar, mental-state language, and conversational appropriateness are typically considered discourse-pragmatic outcomes.

### Structural language and developmental language disorder

DLD is characterized by persistent difficulties in language acquisition, with particular vulnerability in phonological, morphosyntactic, and lexical-semantic domains (Jakubowicz and Tuller, 2007; Leonard, 2014). Although the severity and profile of impairment vary across individuals, children with DLD commonly exhibit co-occurring difficulties in vocabulary, syntax, and phonological learning (Bishop et al., 2016). These deficits are evident across languages and are among the most reliable clinical markers of the disorder.

In the phonological domain, children with DLD often exhibit developmental patterns similar to those of younger typically developing children (Leonard, 1998). They frequently omit or substitute sounds, experience difficulties with consonant clusters and polysyllabic words, and produce speech that may be less intelligible than that of their peers (Aguilar-Mediavilla et al., 2002; Mengisidou et al., 2020).

Deficits in nonword repetition are among the most consistently reported findings in DLD and are considered a robust clinical marker of the disorder. These difficulties are thought to reflect limitations in phonological short-term memory and the encoding of novel phonological forms (Gathercole and Baddeley, 1990; Bishop and Donlan, 1996; Graf Estes et al., 2007; Conti-Ramsden and Hesketh, 2003; Coady and Evans, 2008). Research suggests that children with DLD experience particular difficulty in the initial acquisition and representation of new word forms, although longer-term retention may be relatively preserved (Bishop and Hsu, 2015).

Morphosyntax is one of the most consistently impaired domains in DLD. Children frequently omit grammatical morphemes, particularly those marking tense and agreement, and demonstrate difficulties with a wide range of inflectional forms (Leonard et al., 1992; Redmond, 2005; Krok and Leonard, 2015). Deficits have also been reported in plural marking, possessives, articles, clitics, verb inflection, and aspect marking across a variety of languages (Bedore and Leonard, 1998; Tuller et al., 2011; Chen and Durrleman, 2022).

Children with DLD also show marked difficulties with complex syntactic constructions, particularly those involving noncanonical word order and syntactic movement, such as passives, object relative clauses, and wh-questions (Van der Lely, 1996; Georgiou and Theodorou, 2022). These difficulties have been linked to challenges in processing long-distance dependencies and hierarchical syntactic relations (Friedmann and Novogrodsky, 2004; Friedmann and Haddad-Hanna, 2014).

Sentence repetition has emerged as another robust clinical marker of DLD, with affected children consistently performing below typically developing peers across languages (Conti-Ramsden et al., 2001; Theodorou et al., 2017). Cross-linguistic studies further highlight the consistency of morphosyntactic vulnerability, with deficits reported in clitic production in French, thematic role assignment and pronoun interpretation in Greek, and ba/bei constructions in Mandarin (Stavrakaki, 2001; Stavrakaki and van der Lely, 2010; Yu et al., 2022). Article use appears particularly vulnerable, with frequent omissions and substitutions, especially in definite contexts (Chondrogianni et al., 2015; Andreou and Aslanoglou, 2022; Leonard, 2016; Blom et al., 2015).

Children with DLD typically exhibit weaker lexical knowledge, smaller vocabularies, and slower rates of word learning than their typically developing peers (Adams, 2002; Gray, 2003; Edwards and Lahey, 1996; Kan and Windsor, 2010; Leonard, 2009). Difficulties are particularly evident for abstract, low-frequency, and morphologically complex words (Ellis Weismer and Hesketh, 1996; Dawson et al., 2023).

Lexical representations in DLD are often less detailed and less efficiently organized, resulting in difficulties with semantic encoding, storage, and retrieval (Alt et al., 2013). These weaknesses have been linked to broader limitations in phonological processing, working memory, semantic learning, and procedural learning mechanisms (Alt and Plante, 2006; Collisson et al., 2015; Ullman and Pierpont, 2005). Children with DLD also experience difficulties in literal and inferential comprehension, particularly when tasks place increased demands on vocabulary knowledge and semantic integration (Bishop and Adams, 1992).

Several theoretical accounts have been proposed to explain these difficulties. The Hierarchical Ordering Deficit (HOD) account suggests that children with DLD rely more heavily on linear than hierarchical processing strategies, resulting in difficulties with sentence comprehension and complex grammatical structures (Cromer, 1978). Other accounts emphasize impairments in syntactic movement, long-distance dependencies, and grammatical binding, although findings remain mixed and may be influenced by language-specific characteristics, task demands, and participant heterogeneity.

Narrative discourse is widely regarded as a sensitive measure of structural language ability because it requires the integration of lexical, morphological, and syntactic knowledge in connected speech (Bruner, 1991). Narrative tasks frequently reveal structural language weaknesses in children with DLD that may not be fully apparent in standardized assessments. Compared with typically developing peers, children with DLD produce shorter and less syntactically complex narratives, demonstrate reduced lexical diversity, and exhibit lower grammatical accuracy (Norbury and Bishop, 2003; Manolitsi and Botting, 2011; Leonard, 2014; Colozzo et al., 2011). Difficulties are also evident in referential cohesion, particularly in the use of pronouns and other referring expressions (Fey et al., 2004). Furthermore, increasing syntactic complexity places additional demands on language production, often resulting in higher rates of morphosyntactic errors even when overall narrative content remains relatively preserved (Colozzo et al., 2011). These findings highlight the value of narrative assessment for identifying structural language difficulties in naturalistic communicative contexts.

### Structural language: autism spectrum disorder and developmental language disorder

The linguistic similarities and differences between ASD and DLD have been widely examined in the literature (Brignell et al., 2018; Eigsti and Bennetto, 2009; Tager-Flusberg, 2006; Terzi et al., 2014; Tomblin, 2011; Tuller et al., 2017). Although both conditions are characterized by considerable heterogeneity, research has consistently identified patterns of both overlap and distinction in their language profiles.

Studies focusing on structural language have reported notable similarities between children with ASD and DLD, supporting the existence of a partial phenotypic overlap (Bishop, 2010a,b; Kjelgaard and Tager-Flusberg, 2001; Tager-Flusberg and Joseph, 2003). This overlap is particularly evident among children with ASD and co-occurring language impairment (ASD-LI), who often exhibit morphosyntactic and lexical-semantic profiles comparable to those observed in DLD (Manolitsi and Botting, 2011; McGregor et al., 2012; Riches et al., 2010; Tuller et al., 2017). Consistent with this view, standardized language assessments, such as the Clinical Evaluation of Language Fundamentals (CELF-4), frequently reveal few or no significant differences between children with ASD-LI and those with DLD, suggesting shared structural language vulnerabilities.

Nonword repetition (NWR), a widely used measure of phonological memory and phonological processing, is one of the most frequently examined areas of overlap between ASD and DLD. Children with DLD consistently demonstrate marked impairments on NWR tasks, particularly as phonological complexity and item length increase. Similar, although less consistent, difficulties have been reported in children with ASD and co-occurring language impairment (Graf Estes et al., 2007).

However, findings across studies remain mixed. Some research has reported comparable levels of impairment in children with ASD and DLD (Demouy et al., 2011; Whitehouse et al., 2008), supporting the notion of shared phonological vulnerabilities. In contrast, other studies have found that children with ASD perform significantly better than children with DLD on NWR tasks (Durrleman and Delage, 2016; Riches et al., 2011). These findings suggest that although phonological processing difficulties may be present in both disorders, deficits are generally more pronounced and consistent in DLD, whereas phonological abilities in ASD appear more heterogeneous and are often influenced by the presence or absence of co-occurring language impairment. Consequently, NWR performance provides evidence of both overlap and distinction between the two conditions and may reflect differences in the underlying mechanisms supporting phonological processing.

Morphosyntactic difficulties represent one of the strongest areas of overlap between ASD and DLD. Both groups frequently omit verb tense morphemes—particularly in English—a feature traditionally considered a clinical marker of DLD but also observed in children with ASD and co-occurring language impairment (Demouy et al., 2011; Roberts et al., 2004; Whitehouse et al., 2008). Similar patterns have been reported in article use, with children in both groups overproducing indefinite articles in definite contexts (Creemers and Schaeffer, 2015; Schaeffer et al., 2017). Nevertheless, important differences exist. Children with ASD and typical language abilities often demonstrate relatively intact determiner use, whereas children with ASD-LI show morphosyntactic profiles more comparable to those of children with DLD (Schaeffer, 2018).

Evidence of overlap is also found in clitic production. Children with ASD-LI may exhibit reduced production of third-person accusative clitics similar to that observed in children with DLD (Durrleman and Delage, 2016; Tuller et al., 2017). However, clitic difficulties in ASD have often been linked to challenges at the syntax-pragmatics interface, particularly in the encoding of discourse information, whereas deficits in DLD are more commonly interpreted as reflecting core morphosyntactic weaknesses (Terzi et al., 2014).

In the syntactic domain, both ASD and DLD are associated with difficulties in complex structures involving movement operations, including wh-questions, relative clauses, and passives (Durrleman et al., 2017; Prévost et al., 2017; Riches et al., 2010). Cross-linguistic studies have documented delayed comprehension and production of wh-movement structures in both groups (Goodwin et al., 2012), suggesting shared vulnerability in the processing of syntactically complex constructions.

At the same time, qualitative differences have been reported. While some studies have found comparable performance in grammatical tasks across ASD and DLD (Tuller et al., 2017; Whitehouse et al., 2008), others suggest that language difficulties in ASD may be less selective, affecting both simple and complex structures, whereas DLD is characterized by more consistent impairments in specific grammatical constructions (Durrleman et al., 2016). Furthermore, individuals with ASD are more likely to produce pragmatically inappropriate responses in syntactic tasks, whereas children with DLD typically produce structurally based errors without accompanying pragmatic violations (Sukenik and Friedmann, 2018; Sukenik et al., 2021). Overall, both groups are sensitive to syntactic complexity, although morphosyntactic impairments tend to be more severe and pervasive in DLD.

Findings regarding lexical-semantic abilities in ASD and DLD are less consistent than those reported for phonology and morphosyntax. Some studies have found comparable vocabulary size and lexical performance in the two groups (Demouy et al., 2011; Manolitsi and Botting, 2011; McGregor et al., 2012; Whitehouse et al., 2008), supporting the notion of overlap in lexical-semantic development. However, other studies have reported differences, with children with ASD demonstrating either relative strengths or weaknesses depending on task demands and the presence of co-occurring language impairment (Loucas et al., 2013; Manolitsi and Botting, 2011).

Evidence from both populations points to difficulties in lexical-semantic processing, including reduced depth of word knowledge, weaker semantic representations, and challenges in phonological and semantic encoding (McGregor et al., 2012; Nash and Donaldson, 2005; Steele and Watkins, 2010). Nevertheless, important differences have been reported. Children with DLD tend to show more consistent deficits in semantic organization, lexical access, and word learning (Sheng and McGregor, 2010), whereas lexical-semantic performance in ASD appears more heterogeneous and is often influenced by overall language ability (McGregor et al., 2012; Tager-Flusberg, 1991).

Some studies have further suggested that children with ASD outperform those with DLD on lexical tasks involving word associations, semantic categorization, and structural relations between words (Loucas et al., 2013; Manolitsi and Botting, 2011). Overall, the evidence indicates substantial overlap between ASD and DLD in lexical-semantic development, although deficits tend to be more pervasive and consistent in DLD, whereas lexical abilities in ASD are characterized by greater variability and are strongly influenced by the presence or absence of co-occurring language impairment.

Narrative studies provide further evidence of both overlap and distinction between ASD and DLD. Several studies have reported similar narrative profiles across the two groups, with children with ASD and DLD producing narratives that are less grammatically accurate, less syntactically complex, and less cohesive than those of typically developing peers (Norbury and Bishop, 2003; Norbury et al., 2014). These findings suggest shared weaknesses in the integration of lexical and morphosyntactic knowledge during connected speech production.

At the same time, important differences have been reported. Children with DLD tend to exhibit more consistent deficits in grammatical accuracy, syntactic complexity, and lexical diversity, whereas children with ASD often demonstrate additional difficulties in referential cohesion and narrative organization (Manolitsi and Botting, 2011). Increased linguistic demands frequently lead to higher rates of morphosyntactic errors in both groups, highlighting the sensitivity of narrative tasks for identifying structural language weaknesses that may not be apparent in standardized assessments.

Evidence from language-processing studies suggests that structural language abilities contribute substantially to performance in both ASD and DLD. Difficulties have been reported in lexical-semantic processing, including reduced depth of word knowledge and challenges in phonological and semantic encoding (McGregor et al., 2012; Nash and Donaldson, 2005; Steele and Watkins, 2010). Both groups also demonstrate difficulties with vocabulary learning, although orthographic support may facilitate lexical acquisition and retention (Ricketts, 2011).

Studies examining context-sensitive language comprehension indicate that structural language ability is a stronger predictor of performance than diagnostic status alone. Children with ASD and DLD who have weaker structural language skills show similar difficulties in integrating contextual information during language comprehension, whereas those with stronger language abilities perform more successfully (Norbury, 2005). These findings suggest that many apparent differences in language processing may reflect underlying variation in structural language abilities rather than disorder-specific mechanisms.

### Overlap vs. distinction

Overall, the literature suggests both overlap and distinction in the language profiles of children with ASD and DLD. Similarities are most evident in structural language domains, particularly phonology, morphology, and syntax, where children with ASD and ASD-LI often perform similarly to children with DLD. However, these similarities do not necessarily imply common underlying mechanisms. DLD is primarily characterized by persistent deficits in structural language, particularly morphosyntax and phonological processing, whereas language profiles in ASD are more heterogeneous and are frequently influenced by broader cognitive, social, and pragmatic factors.

Although some researchers have proposed a continuum between ASD and DLD (Oi et al., 2017), others argue that the observed similarities reflect partially overlapping phenotypes that arise from distinct developmental and etiological pathways (Williams et al., 2008). Evidence from nonword repetition, lexical-semantic processing, morphosyntactic tasks, and narrative measures of structural language suggests that ASD and DLD share certain linguistic vulnerabilities while also exhibiting disorder-specific patterns of impairment (Creemers and Schaeffer, 2016a,b; Whitehouse et al., 2008).

The present systematic review aims to synthesize empirical evidence on structural language abilities in children with ASD and DLD. Specifically, it examines similarities and differences across the domains of phonology, morphology, syntax, and semantics, as well as narrative measures that reflect the integration of these components in connected speech. By organizing findings according to these domains, the review seeks to identify areas of convergence and divergence and to evaluate whether observed similarities reflect shared linguistic vulnerabilities or distinct underlying processes.

The review also considers heterogeneity within each condition, including subgroups such as children with ASD and ASD-LI, as well as methodological factors that may contribute to variation across studies, including assessment tools, task demands, and participant characteristics. By integrating findings across structural language domains, this review aims to provide a comprehensive understanding of language phenotypes in ASD and DLD, clarify the nature of their relationship, and contribute to ongoing theoretical debates regarding language development in neurodevelopmental disorders. The findings may also inform clinical assessment and differential diagnosis and support the development of more targeted language interventions for both populations.

Accordingly, the following research questions were formulated to guide the present systematic review:

(a) To what extent do children with Autism Spectrum Disorder (ASD) and Developmental Language Disorder (DLD) demonstrate similar structural language profiles across the domains of phonology, morphology, syntax, semantics, and narrative language?
(b) Which structural language characteristics are shared between ASD and DLD, and which features differentiate the two disorders?
(c) Do the observed similarities between ASD and DLD reflect a common structural language phenotype, or do they arise from distinct underlying linguistic and cognitive mechanisms?

By addressing these questions, the review aims to clarify the nature of structural language impairment in ASD and DLD, identify areas of convergence and divergence across language domains, and contribute to the ongoing debate regarding the relationship between the two disorders.

## Materials and methods

### PICOS eligibility criteria

The eligibility criteria were defined according to the PICOS framework as follows:

- Population (P): Children aged approximately 3–18 years diagnosed with Autism Spectrum Disorder (ASD) and Developmental Language Disorder (DLD), including studies using earlier diagnostic labels such as Specific Language Impairment (SLI), Language Impairment (LI), Expressive Language Disorder (ELD), or Pragmatic Language Impairment (PLI), where appropriate.
- Intervention/Exposure (I): Assessment of structural language abilities, including phonology, morphology, syntax, semantics, and narrative measures reflecting structural language performance.
- Comparator (C): Direct comparisons between children with ASD and DLD or equivalent language-disorder groups. Studies including typically developing control groups were also eligible when they reported direct comparisons between ASD and DLD.
- Outcomes (O): Structural language performance measured using standardized language assessments, experimental linguistic tasks, or language sample analyses assessing one or more structural language domains.
- Study design (S): Empirical, peer-reviewed studies employing cross-sectional, longitudinal, experimental, observational, or comparative designs, published in English between 2013 and 2025.

### Eligibility criteria

Studies were included in the present systematic review if they met the following criteria:

(1) empirical studies published in peer-reviewed journals in English;
(2) studies including children diagnosed with ASD and DLD within the age range of approximately 3 to 18 years;
(3) studies examining structural language domains, including phonology, morphology, syntax, semantics, or narrative measures reflecting structural language abilities;
(4) studies that directly compared ASD and DLD groups or provided data allowing comparison across these populations;
(5) studies in which assessment procedures, linguistic measures, and tasks were clearly described;
(6) studies published between 2013 and the 2025.

Studies were excluded if they:

(1) Focused solely on the development or validation of assessment tools without reporting empirical findings;
(2) Included participants without a formal diagnosis of ASD or DLD;
(3) Examined language abilities outside the scope of structural language;
(4) Were doctoral dissertations, theses, conference abstracts, book chapters, reviews, or other non-peer-reviewed publications;
(5) Included populations outside the scope of the review (e.g., Social (Pragmatic) Communication Disorder), unless data for ASD and DLD were reported separately.

Across included studies, ASD diagnoses were based on established diagnostic criteria, including the Diagnostic and Statistical Manual of Mental Disorders (DSM-IV or DSM-5) or the International Classification of Diseases (ICD-10), and included subtypes such as Autistic Disorder, Asperger Syndrome/High-Functioning Autism, and Pervasive Developmental Disorder-Not Otherwise Specified. DLD/SLI diagnoses were established using standardized language assessments administered by qualified professionals and were defined as persistent language difficulties not attributable to intellectual disability, hearing impairment, neurological conditions, or other known biomedical causes. Where reported, participants demonstrated nonverbal cognitive abilities within the normal range, ensuring that language difficulties were not secondary to broader cognitive impairments. Because the included studies spanned the period before and after the publication of the CATALISE recommendations (Bishop et al., 2017), original diagnostic labels (e.g., SLI, LI, ELD, PLI) were retained when reporting individual studies. For the purposes of synthesis and discussion, these labels were interpreted within the broader DLD framework where appropriate, while preserving the terminology used by the original authors. Likewise, original ASD subgroup labels (e.g., ASD-LI, ASD-LN, HFA-LI, and related classifications) were retained throughout the review. Where comparisons across studies were made, findings were synthesized according to the structural language characteristics of the reported participant groups while acknowledging differences in diagnostic terminology and subgroup definitions across studies.

### Search procedure

This systematic review was conducted in accordance with the Preferred Reporting Items for Systematic Reviews and Meta-Analyses (PRISMA) 2020 guidelines (Page et al., 2021). The final literature search was conducted on 12 November 2025.

A comprehensive search was conducted in the primary bibliographic databases PubMed (NCBI), ERIC, and APA PsycInfo (via PsycNet). Google Scholar and Semantic Scholar were used only as supplementary sources to identify additional potentially relevant studies through citation searching and broader literature retrieval. Because these platforms have limited reproducibility and transparency for systematic searching, they were not treated as primary bibliographic databases. The reference lists of eligible articles were also manually screened to identify additional relevant studies.

The search strategy was developed using combinations of controlled vocabulary (where available) and free-text keywords related to Autism Spectrum Disorder (ASD), Developmental Language Disorder (DLD)/Specific Language Impairment (SLI), and structural language domains. Searches combined terms for the target populations, structural language characteristics, and relevant age groups using Boolean operators (AND, OR). Search syntax was adapted to the indexing system and search functionality of each database while maintaining the same conceptual framework. The complete database-specific search strategies, including the exact search strings and search limits, are provided in Supplementary Table S1.

Searches were limited to English-language, peer-reviewed studies published between 1 January 2013 and 12 November 2025.

### Study selection procedure

All records identified through the search strategy were imported into the review database. Duplicate records were identified and removed prior to screening. The remaining records underwent title screening by the lead author to identify studies potentially relevant to the review topic. Studies that were clearly unrelated to the aims of the review were excluded at this stage.

Subsequently, the abstracts of the remaining studies were screened independently by all authors according to the predefined inclusion and exclusion criteria. Studies that did not meet the eligibility criteria were excluded. Full-text versions of potentially eligible articles were then retrieved and assessed independently by both authors for inclusion. Studies that failed to meet the inclusion criteria were excluded, and the reasons for exclusion were documented. Any disagreements or uncertainties regarding study eligibility were resolved through discussion until consensus was reached. Data extraction was performed by the lead author using a standardized data extraction form and independently reviewed by the second author for accuracy and completeness. Quality appraisal was performed independently by both authors, with any discrepancies resolved through discussion until consensus was reached. The final set of studies included in the review was determined following full-text assessment and was subsequently included in the qualitative synthesis.

**Figure 1:**
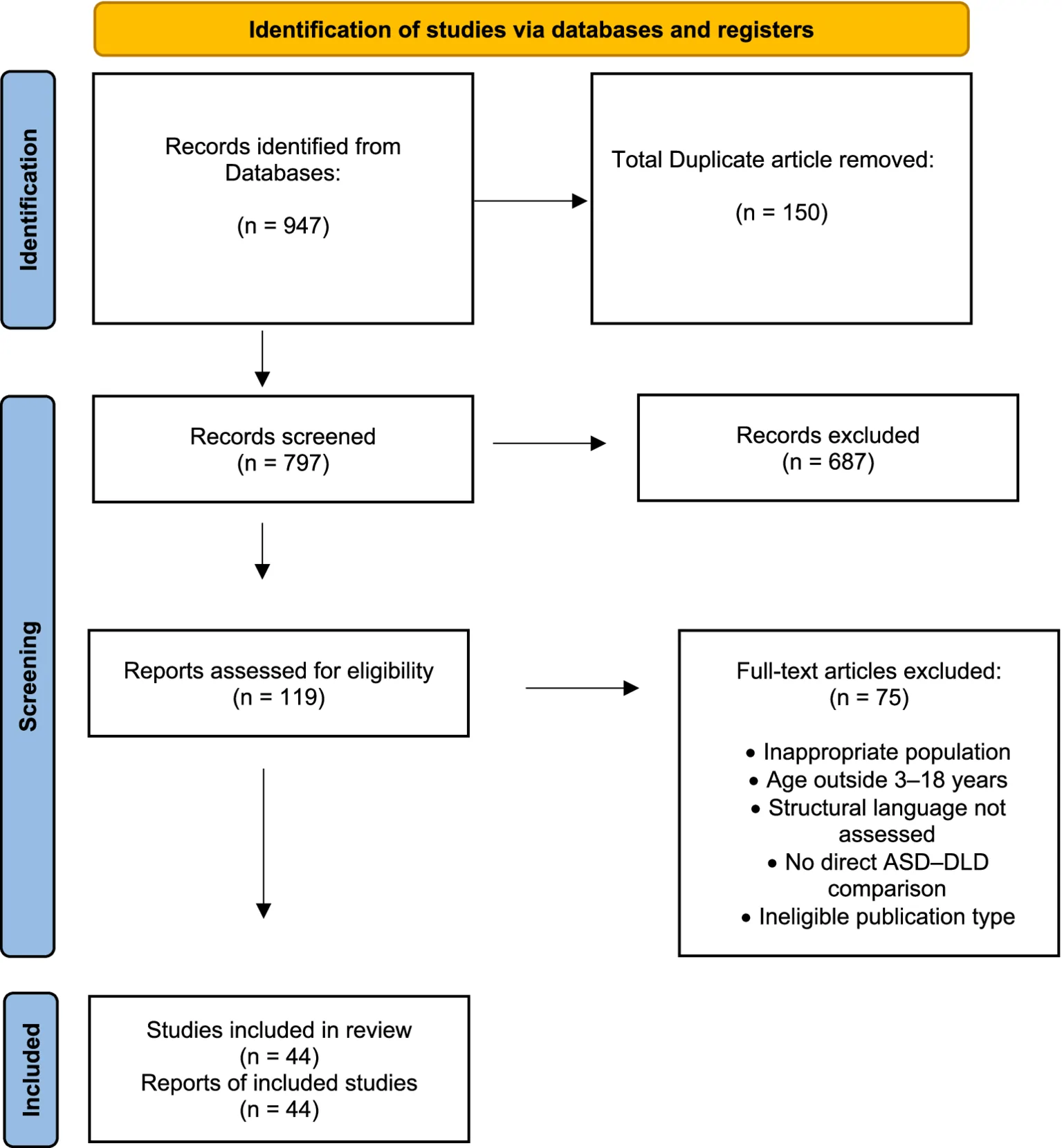
PRISMA 2020 flow diagram of the identification, screening, eligibility assessment, and inclusion of studies.

A total of 947 records were identified through database searching. After duplicate records were removed, 797 records remained for screening. During title screening, 455 records were excluded because they were clearly irrelevant to the review topic. The remaining 342 records underwent abstract screening. Following abstract review, 223 records were excluded for not meeting the inclusion criteria, leaving 119 full-text articles for eligibility assessment. Of these, 75 articles were excluded for reasons including an inappropriate study population, participant age outside the predefined age range, failure to assess structural language domains, lack of a direct comparison between ASD and DLD, or publication type not meeting the inclusion criteria. A total of 44 studies met all eligibility criteria and were included in the qualitative synthesis. PRISMA 2020 flow diagram illustrating the study selection process (Figure 1).

### Data extraction

For each included study, the following information was extracted: author(s), year of publication, country, study design, participant characteristics (age, sample size, and diagnostic group), language domains assessed, assessment tools, and main findings.

Given the heterogeneity of study designs, languages, and outcome measures, a narrative synthesis approach was adopted. Findings were organized according to structural language domains - phonology, morphology, syntax, semantics, and narrative measures of structural language - to facilitate systematic comparison between ASD and DLD. A domain-based framework was selected because these components represent core aspects of linguistic competence and are central to the characterization of language phenotypes in neurodevelopmental disorders. Organizing findings in this way enabled evaluation of whether similarities between ASD and DLD reflected shared structural language vulnerabilities or distinct underlying mechanisms. Where narrative tasks were used, findings were classified according to the structural language components assessed (e.g., lexical diversity, grammatical accuracy, mean length of utterance, and syntactic complexity) rather than broader discourse-pragmatic outcomes The characteristics and main findings of the included studies are presented in Table 1.

**Table 1:** Studies included in the review.

Author (s), year	Country	Study design	Age (mean/range)	Participants (n)	Language domain(s) assessed	Assessment tools	Main findings
Ramírez-Santana et al. (2019)	Spain	Cross-sectional	7.0–11.5 years	*N* = 60 (ASD = 20, SLI = 20, TD = 20)	Language Structure (sentence comprehension, sentence production, auditory memory), Language Content (vocabulary and semantic associations), Receptive and Expressive Language	CELF-4 standardized test	No significant differences were found between the ASD and SLI groups across language measures; both groups performed significantly below controls, showing highly overlapping structural language profiles
Georgiou and Spanoudis (2021)	Cyprus	Cross-sectional, experimental	6–12 yrs.	ASD (*n* = 40), DLD (*n* = 28), TD (*n* = 35)	Receptive and expressive language, morphology, syntax, semantics/vocabulary, phonological memory, communication/pragmatic abilities	EREL (Concepts and Following Directions, Word Structure, Formulated Sentences, Recalling Sentences, Word Classes), Non-Word Repetition (NWR), CCC-2	Identified an ASD-LI subgroup with language profiles similar to DLD; both ASD-LI and DLD performed below TD on language measures.
Doghadze and Gagoshidze (2023)	Georgia	Cross-sectional comparative study	Age 4–6	ASD (*n* = 45), ELD (*n* = 48), TLD (*n* = 55)	Phonology, semantics, syntax, morphology, pragmatics; functional language (mands, tacts, echoics, intraverbals)	Structured speech sampling, picture description tasks, spontaneous conversation; linguistic error analysis; verbal behavior framework (Skinner)	Children with DLD showed predominant phonological and morphosyntactic errors, whereas children with ASD demonstrated greater pragmatic difficulties. Both clinical groups differed significantly from TLD, supporting distinct language profiles.
Brignell et al. (2018)	Australia	Longitudinal (prospective cohort study)	4 to 7 years	ASD (*n* = 27), LI (*n* = 119), TD (*n* = 861)	Structural language: Morphology Syntax Receptive and expressive language	CELF-P2, CELF-4 (receptive & expressive indices) KBIT-2/WASI (IQ)	ASD and LI showed comparable structural language deficits and performed below TD peers. Language outcomes were associated with early language ability and IQ rather than diagnosis.
Oi et al. (2017)	Japan	Large national population-based survey with clinical comparison groups	3–15 years	ASD (*n* = 48), LI (*n* = 30), TD (*n* = 64)	Structural language: speech, syntax, semantics, coherence; also pragmatic/social communication domains	Japanese Children’s Communication Checklist-2 (CCC-2); ADOS-G and DISCO for ASD diagnosis; WISC-III for LI group IQ; SCQ and LDI-R for LI screening	ASD and LI showed comparable structural language deficits (speech, syntax, semantics, coherence) below TD levels. ASD exhibited greater social-pragmatic impairment.”
Andreou et al. (2025)	Greek	Cross-sectional quantitative comparative study	6–8 years	ASD (*n* = 25), DLD (*n* = 25), TLD (*n* = 25)	Phonological awareness, morphosyntax, expressive vocabulary	Greek version of the Children’s Communication Checklist (CCC-2)	TD children outperforming both clinical groups, DLD showing the most severe impairments, and ASD demonstrating intermediate performance, while expressive vocabulary scores were also higher in the ASD group.
Dockrell et al. (2019)	United Kingdom	Longitudinal observational study (two time points)	Mean age = 10.2 years (6–12 years)	*N* = 157 (DLD = 93, ASD = 64)	Structural language (expressive and receptive) Literacy (reading, comprehension, spelling) Social communication/pragmatics	CELF-4 (Clinical Evaluation of Language Fundamentals) BAS-II (cognitive ability, spelling) YARC (reading comprehension) SWRT (single-word reading) SRS (Social Responsiveness Scale) SDQ (Strengths and Difficulties Questionnaire)	DLD and ASD showed similar structural language deficits, although DLD exhibited more severe language, literacy, and spelling impairments. ASD demonstrated greater social and behavioral difficulties.
Gibson et al. (2013)	UK	Comparative cross-sectional study	6–11 years	HFA (*n* = 21), PLI (*n* = 22), SLI (*n* = 19)	Expressive & receptive language; pragmatic/social communication	CELF-4, CCC-2, ADOS-G, SCQ, MIPO, RBQ-2	HFA showed stronger structural language abilities than SLI and PLI, which exhibited persistent expressive language deficits.
Hare-Harris et al. (2019)	United States	Retrospective cross-sectional study	Mean age ≈ 13 years	*N* = 187 (ASD = 17, LI = 28, ASD + LI = 27, TD = 115)	Morphology, syntax, expressive language, receptive language, semantics, metalinguistic language, pragmatic language	Clinical Evaluation of Language Fundamentals–4 (CELF-4); Comprehensive Assessment of Spoken Language (CASL); Social Responsiveness Scale (SRS)	Standard language measures did not reliably distinguish LI from ASD. ASD groups showed greater language variability, which was associated with ASD traits and diagnosis
Taylor et al. (2014)	Australia	Cross-sectional comparative study	Age range: 5.0–12.7 years	*N* = 112 (TD = 61, ASD-LN = 18, ASD-LI = 14, SLI = 19)	Phonology; phonological short-term memory; nonword repetition; sentence repetition (repetition-based markers of structural language impairment)	TROG-2; NEPSY-II Nonword Repetition; NEPSY-II Sentence Repetition; WASI Matrix Reasoning; ADOS-G; CCC-2; Children’s Embedded Figures Test	ASD-LI and SLI showed similar repetition deficits, whereas ASD without language impairment performed comparably to TD peers
Hill et al. (2015)	USA	Cross-sectional comparative study	5–8 years;	SLI (*n* = 18), ASD + LI (*n* = 22), ASD-LN (*n* = 20)	Phonology; nonword repetition; verbal memory	Nonword Repetition Test (NWR), CELF, digit span, narrative memory	ASD + LI and SLI showed impaired nonword repetition and verbal memory; SLI exhibited more severe phonological memory deficits, while ASD-LN performed best
Knowland et al. (2022)	United Kingdom	Experimental ERP study	Age range: 7.11–13 years	*N* = 56 (ASD = 19, DLD = 16, TD = 21)	Phonological representations, novel word learning, phonological processing, lexical encoding and consolidation	ERP auditory N400 paradigm; pseudoword and word repetition tasks; BPVS-3; CELF Recalling Sentences; CTOPP-2 RAN	DLD showed weaker neural responses and reduced phonological consolidation, whereas ASD demonstrated phonological learning patterns similar to TD peers.
Dynia et al. (2019)	United States	Longitudinal cohort study (fall–spring preschool year)	Age range: 4–5 years	N = 125 (ASD = 27, LI = 28, TD = 70)	Phonological awareness (syllable awareness, onset-rime awareness, blending, elision, receptive and expressive phonological awareness)	Test of Preschool Early Literacy (TOPEL) – Phonological Awareness Subtest; CELF-Preschool; KBIT; SSRS	ASD and LI showed lower phonological awareness than TD peers, with persistent deficits across both clinical groups.
Chen et al. (2022)	China	Cross-sectional, group-comparative	Age range: 4–6 years	*N* = 60 (HFA-LI = 20, DLD = 20, TDA = 20)	Grammatical morphology (aspect markers), comprehension & production	Picture-choice task; Priming picture-description task; PPVT-R; RSPCLD-R; WPPSI-IV	HFA-LI and DLD showed similar deficits in aspect-marker comprehension and production, supporting overlap in grammatical morphology
Prévost et al. (2018)	France	Cross-sectional comparative study (elicited production task)	Age range: 6–12 years	*N* = 38 (ASD = 19 [ASD-LI = 14, ASD-LN = 5], SLI = 19)	Morphosyntax Morphosyntax (pronominal clitic production), grammatical complexity, pronoun use	Production Probe for Pronoun Clitics (PPPC); standardized language tests of phonology, morphosyntax, and vocabulary	ASD-LI and SLI showed similar clitic production deficits, particularly for third-person accusative clitics, indicating shared structural impairment
Durrleman and Delage (2016)	Switzerland/France	Cross-sectional comparative experimental study	Age range: 5–16 years	N = 87 (ASD = 21, SLI = 22, TD = 44)	Morphosyntax, expressive grammar, pronominal clitic production (1st- and 3rd-person accusative clitics), syntax–working memory relations	Production Probe for Pronoun Clitics (PPPC); BILO Expressive Grammar Test; Nonword Repetition; Digit Span; Raven’s Progressive Matrices; Sally-Anne ToM Task	ASD and SLI showed comparable morphosyntactic and clitic production deficits. ASD exhibited greater heterogeneity and additional first-person clitic difficulties.
Zhang et al. (2024)	China	Cross-sectional experimental study	Mean age ≈ 5.3 years	N = 88 (DLD = 28, ALI = 32, TD = 28)	Morphosyntax –semantics interface, definiteness marking, pronoun comprehension, DP (Determiner Phrase) interpretation	Picture Judgment Tasks assessing demonstrative-classifier NPs, third-person pronouns, and bare NPs in anaphoric contexts	DLD and ALI showed similar deficits in definiteness processing, although ALI demonstrated greater difficulty with third-person pronoun comprehension
Schaeffer (2018)	Netherlands	Cross-sectional, experimental comparative study	Range: 5–14 years	*N* = 81 (HFA = 27, SLI = 27, TD = 27)	Morphosyntax (mass-count distinction, subject–verb agreement), Pragmatics (article choice, direct object scrambling)	Quantity Judgment Task (Mass–Count); Subject–Verb Agreement Elicited Production Task; Article Choice Elicited Production Task; Direct Object Scrambling Task; CELF-4-NL Sentence Repetition	SLI showed significant morphosyntactic deficits, whereas HFA performed at TD levels and outperformed the SLI group.
Ellis Weismer et al. (2017)	USA	Cross-sectional, comparative study	Mean age = 9.5–9.9 years	*N* = 84 (TD = 36, ASD = 27, SLI = 21)	Morphosyntax (grammatical morphology; morphosyntactic processing)	Grammatical Judgment Task (omission of past tense -ed and auxiliary markers); Visual N-back Task (nonverbal working memory); CELF-4	Children with language impairment showed poorer morphosyntactic processing regardless of diagnosis; performance was associated with nonverbal working memory.
Huang and Finestack (2020)	United States	Cross-sectional comparative study	Age range: 4.4–9.8 years	N = 36 (DLD = 21, LD-ASD = 15)	Morphology and Syntax (Morphosyntax)	Structured Photographic Expressive Language Test–Third Edition (SPELT-3); Index of Productive Syntax (IPSyn) based on language sample analysis	DLD and LD-ASD showed highly similar morphosyntactic profiles, with only minor differences across specific grammatical structures.
Prévost et al. (2017)	France	Cross-sectional comparative study	Age range: 6.3–12.9 years	*N* = 69 (ASD = 20, SLI = 20, TD = 29)	Morphosyntax (wh-question production and comprehension), complex syntax, syntactic complexity	Elicited Production Task for French *wh*-questions; *wh*-Question Comprehension Task; standardized tests of phonology, morphosyntax, vocabulary, and nonverbal reasoning (RPM)	ASD and SLI showed similar difficulties with complex wh-questions, indicating substantial overlap in morphosyntactic impairment.
Nie et al. (2025)	China	Cross-sectional comparative study	Mean ages: DLD = 5.3, ALI = 5.5, TD = 5.2 years	*N* = 59 (DLD = 18, ALI = 17, TD = 24)	Syntax/Morphosyntax (complex sentence comprehension and production; Mandarin *ba* and *bei* constructions; non-canonical word order)	Sentence-Picture Matching Task; Priming Picture-Description Task; WPPSI-IV NVWM measures (Picture Memory, Zoo Locations)	DLD and ALI showed similar deficits in ba/bei constructions, but DLD produced more syntactic errors whereas ALI showed more pragmatic errors.
Dai et al. (2025)	China	Cross-sectional comparative study	Mean ages: DLD = 5.1 years, ALI = 5.4 years, TD = 5.2 years	*N* = 56 (DLD = 15, ALI = 16, TD = 25)	Syntax / Morphosyntax (passive constructions, wh-question comprehension, movement and intervention effects)	Picture-choice task; Character-picture matching task assessing comprehension of Mandarin passive clauses and wh-questions	DLD and ALI showed similar difficulties with complex passives and wh-questions, supporting overlap in morphosyntactic impairment.
Fortunato-Tavares et al. (2015)	Brazil	Cross-sectional comparative study	Age range: 7.0–14.2 years	*N* = 57 (SLI = 15, HFA = 12, DS = 15, TLD = 15)	Syntax / Morphosyntax, sentence comprehension, predicate attachment, reflexive pronoun assignment, syntax–working memory relations	Computerized Picture-Selection Sentence Comprehension Task (Predicate Attachment and Reflexive Assignment Tasks with short and long working-memory conditions)	HFA and SLI showed similar syntactic comprehension deficits, with working memory contributing to performance, particularly in SLI.
Moreno-Pérez et al. (2020)	Spain	Cross-sectional experimental study (online eye-tracking + offline comprehension tasks)	Ages: ASD ≈ 12 years (6–18), DLD ≈ 10.4 years (7–14)	*N* = 73 (ASD-HL = 15, ASD-LL = 13, DLD = 15, TD = 15, Age-matched TD = 15)	Morphosyntax/Sentence Comprehension (pronoun interpretation, reflexive pronouns, clitic pronouns, anaphoric reference resolution)	CEG (Grammar Structure Comprehension Test), Eye-tracking pronoun processing task, Offline pronoun comprehension/acceptability judgment task, WISC-IV/WAIS-III	Reflexive and clitic pronoun comprehension did not differentiate ASD and DLD; ASD showed longer reaction times, indicating increased processing effort.
Sukenik and Friedmann (2018)	Israel	Cross-sectional comparative experimental study	Age range: 5.2–18.1 years	*N* = 277 (ASD = 18, SyDLI = 93, TD = 166)	Syntax/Morphosyntax (relative clauses, Wh-questions, topicalization, sentence repetition, complex sentence comprehension and production)	Relative Clause Elicitation Task; Reading and Paraphrasing of Object Relatives; Sentence Repetition Task	ASD and SyDLI showed similar overall syntactic performance, but SyDLI exhibited selective syntactic deficits, whereas ASD showed greater heterogeneity and pragmatic/discourse-related errors.
Sukenik et al., 2021	Israel & France	Cross-sectional comparative study	Age range: 6.3–18.0 years	*N* = 82 (ASD-FR = 20, ASD-HE = 18, DLD-FR = 20, TD-FR = 12, TD-HE = 12)	Syntax / Morphosyntax (*wh*-question production), complex syntax, question formation; pragmatic aspects of question production	Elicited *wh*-Question Production Task (subject and object *wh*-questions); morphosyntactic assessments (sentence completion, sentence repetition)	ASD and DLD showed similarly reduced wh-question accuracy; DLD produced mainly syntactic errors, whereas ASD showed more pragmatic errors
Azevedo et al. (2025)	Portugal	Nonrandomized controlled trial	Age range: 3–6 years	*N* = 31 (DLD = 19, ASD = 12);	Syntax (expressive and receptive syntax)	SIN: TACS (Teste de Avaliação da Competência Sintática) – expressive syntax; Subtest 3 of ALO (Avaliação da Linguagem Oral) – receptive syntax	PROsyntax intervention significantly improved expressive and receptive syntax in both DLD and ASD, with particularly strong gains in ASD.
Durrleman et al. (2019)	Switzerland and France	Intervention study (syntactic training vs. lexical training)	Mean ages: ASD = 8.3, DLD = 6.9, TD = 4.3 years	*N* = 60 (ASD = 16, DLD = 20, TD = 24)	Morphosyntax; complex syntax; sentential complements	False Complement Task; EXALANG Morphosyntax; ToM tasks (verbal and non-verbal false belief)	Complementation training improved complement-clause comprehension and false-belief reasoning in both ASD and DLD, with similar treatment response across groups.
Durrleman et al. (2017)	France/Switzerland	Cross-sectional experimental study (comparative groups: ASD, SLI, TD)	Mean ages: SLI = 9.2, ASD = 10.5, TD = 8.0 years	*N* = 84 (SLI = 20, ASD = 34, TD = 30)	Syntax (complex syntax); sentential complements; complementation	Communication Complement Task (De Villiers and Pyers, 2002); Perception Complement Task (Poltrock, 2010); BILO-3C Morphosyntax and Lexicon Measures; Low-verbal Theory of Mind Task	Children with SLI and ASD showed similar complement-clause comprehension and Theory of Mind performance, highlighting complex syntax as a shared mechanism
Garrido et al. (2024)	Spain	Cross-sectional comparative study	Matched by receptive vocabulary age	*N* = 84 (ALI = 21, ALN = 21, DLD = 21, TD = 21)	Syntax/Morphosyntax (Receptive Grammar)	Grammatical comprehension tasks assessing simple (canonical/non-reversible) and complex (non-canonical/reversible) sentence structures	ALI and DLD showed similar difficulties with complex grammatical structures, indicating overlapping receptive grammar deficits.
Issa et al. (2023)	Jordan	Cross-sectional experimental comprehension study	Age range: 6–8 years	*N* = 45 (ASD = 15, SDLI = 15, TD = 15)	Syntax (wh-question comprehension; syntactic movement/A′-dependencies)	Binary picture-selection tasks (two-figure and three-figure tasks) assessing comprehension of who-subject, who-object, which-subject, and which-object questions	Both ASD and SDLI showed significant wh-question comprehension deficits, although SDLI outperformed ASD on complex wh-dependencies.
Haebig et al. (2015)	USA	Cross-sectional comparative study	Mean ages: 9.1–10.0 years	*N* = 85 (TD = 30, ASD = 27, SLI = 28)	Lexical-semantic processing, vocabulary depth, semantic network organization	Lexical Decision Task (high vs. low semantic network words), PPVT-4, CELF-4, executive function tasks	ASD and SLI showed similar lexical-semantic processing, although semantic network effects were weaker in SLI. Executive functions predicted performance across groups.
Ricketts et al. (2015)	United Kingdom	Cross-sectional comparative study	Age range: 8–13 years	N = 81 (SLI = 27, ASD = 27, TD = 27)	Semantics/vocabulary; spoken word learning; phonology–semantic mapping; orthographic support for vocabulary learning	Experimental nonword–referent learning task; CELF-4 UK Recalling Sentences and Word Classes; WASI Vocabulary; TOWRE word and nonword reading; SRS	ASD, SLI, and TD groups showed similar novel vocabulary learning and benefited from orthographic support; ASD showed slower lexical access, while SLI had weaker baseline vocabulary.
Haebig et al. (2017)	USA	Cross-sectional comparative study	Age range: 8–12 years	*N* = 74 (TD = 26, ASD = 25, SLI = 23)	Lexical-semantic development; word learning; vocabulary acquisition; phonological–semantic mapping	Word Segmentation Task (statistical learning), Fast-Mapping Task, Combination Task (mapping meaning onto newly segmented words), PPVT-4, CELF-4	SLI showed weaker statistical learning and fast mapping than ASD and TD peers; word-learning abilities were relatively preserved in ASD.
Walenski and Love (2017)	USA	Cross-sectional experimental study (cross-modal priming)	Mean ages: TD = 10.6, SLI = 12.2, ASD = 11.5 years	*N* = 26 (TD = 14, SLI = 7, ASD = 5)	Semantic language/lexical-semantic processing, figurative language comprehension (idioms), semantic memory	Cross-Modal Lexical Priming Task (CMLP); CELF-IV; PPVT-IV; TROG-2; CCC-2; TONI-3	SLI and TD showed typical idiom processing, whereas ASD demonstrated a literal interpretation bias and reduced idiomatic meaning activation.
Gladfelter et al., 2019	United States	Cross-sectional experimental word-learning study	Mean age range: 5.1–7.9 years	*N* = 36 (ASD = 12, DLD = 12, TD = 12)	Semantics/Lexical-Semantic Processing, Word Learning, Semantic Representations	Extended novel word-learning paradigm; semantic feature analysis of children’s novel word definitions	ASD and DLD showed similar semantic learning patterns and benefited from visual support during word learning.
Norbury et al. (2014)	United Kingdom & United States (multi-site study)	Cross-sectional comparative study	Age range: 6.5–15 years	*N* = 75 (ASD = 25, LI = 23, TD = 27)	Narrative language, lexical-semantic diversity, syntax, narrative macrostructure, pragmatics	Wordless picture-book narrative task (*A Boy, a Dog and a Frog*), SALT transcription and narrative analyses, CELF-4	ASD and LI showed similar narrative microstructure deficits, while ASD exhibited greater pragmatic difficulties, particularly referential errors.
Govindarajan and Paradis (2022)	Canada	Cross-sectional comparative study	Age range: 5.3–9.1 years	*N* = 29 (ASD = 9, DLD = 10, TD = 10)	Narrative language (macrostructure and microstructure); specifically syntax, lexical diversity, utterance length, story grammar, discourse organization	Edmonton Narrative Norms Instrument (ENNI); Story Grammar Analysis; Mean Length of Communicative Unit (MLCU); Syntactic Complexity Index; Lexical Diversity (NDW); Story Length; PPVT-III; CMMS	ASD and DLD showed similar narrative language deficits, although ASD demonstrated greater difficulties with perspective-taking in narratives.
Nielsen et al. (2025)	Denmark	Cross-sectional comparative study	Age range: 10.5–14.1 years	*N* = 73 (ASD = 28, DLD = 12, TD = 33)	Semantics (intentionality attribution), Narrative discourse (macrostructure)	Social Attribution Task (SAT; Heider & Simmel animation), Physical Attribution Task (PAT), Relevance Index, PPVT, TROG-2, Sentence Repetition, Picture Naming, Digit Span	Both ASD and DLD showed narrative macrostructure difficulties, producing fewer relevant content elements than TD peers; intentionality attribution was preserved in ASD.
Andrés-Roqueta and Katsos (2020)	Spain	Cross-sectional comparative study	Age range: 4–10 years	*N* = 80 (ASD = 20, DLD = 20, Age-matched TD = 20, Language-matched TD = 20)	Structural language (grammar and vocabulary), linguistic pragmatics, social pragmatics, Theory of Mind	CEG (receptive grammar); ELI battery (expressive grammar, receptive and expressive vocabulary); Cavegirl Task (linguistic pragmatics); Strange Stories Task (social pragmatics); False Belief Tasks for ToM	ASD and DLD showed similar linguistic-pragmatic performance when matched for language level, but ASD exhibited greater social-pragmatic difficulties related to Theory of Mind.
Eberhardt and Nadig (2016)	Experiment 1: USA; Experiment 2: Germany	Cross-sectional experimental study	Age range: 8–15 years	*N* = 121; Exp. 1: ASD-NL = 19, TD = 19; Exp. 2: ASD = 30, LI = 24, TD = 30	Semantics; language comprehension; lexical ambiguity resolution; context-sensitive language processing	Homonym picture-selection task, sentence-completion task, CELF-4, TROG-D, PPVT	Structural language ability, rather than ASD diagnosis, predicted context-sensitive language comprehension; ASD with language impairment and LI showed similar deficits
Schaeffer et al. (2017)	Netherlands	Cross-sectional comparative study	Age range: 6–14 years	*N* = 84 (HFA = 28, SLI = 28, TD = 28)	Morphosyntax; syntax–semantics–pragmatics interface; object placement; definiteness/referentiality	Direct Object Scrambling (DOS) Elicited Production Task; CELF-IV; Nonword Repetition; CCC-2; Subject–Verb Agreement Task; Mass–Count Distinction Task	Both HFA and SLI showed difficulties with referential object scrambling; deficits were morphosyntactic in SLI and integration-related in HFA.
Huang et al. (2025)	China	Cross-sectional comparative study	Age range: 4–6 years	*N* = 109 (ALI/ASD = 32, DLD = 21, TD = 28, Adult controls = 28)	Structural language (syntax–semantics), grammatical processing, prosody, interpretation of non-interrogative wh-words	Question–Statement Task; PPVT-R Chinese Version; RSPCLD-R/RSSCLD-R; WPPSI-IV CN	ALI and DLD showed similar syntactic-semantic processing deficits, but only ALI exhibited additional prosodic-processing difficulties.

Characteristics and main findings of studies comparing structural language abilities in children with Autism Spectrum Disorder (ASD) and Developmental Language Disorder (DLD). ASD, Autism Spectrum Disorder; ASD-LI, Autism Spectrum Disorder with Language Impairment; ASD-LN, Autism Spectrum Disorder with Normal Language; ASD-NL, Autism Spectrum Disorder with Normal Language; ASD-HL, Autism Spectrum Disorder with Higher Language Abilities; ASD-LL, Autism Spectrum Disorder with Lower Language Abilities; ALI, Autism with Language Impairment; ALN, Autism with Normal Language; DLD, Developmental Language Disorder; SLI, Specific Language Impairment; LI, Language Impairment; PLI, Pragmatic Language Impairment; ELD, Expressive Language Disorder; LD-ASD, Language Disorder Associated with Autism Spectrum Disorder; HFA, High-Functioning Autism; HFA-LI, High-Functioning Autism with Language Impairment; SyDLI, Syntactic Developmental Language Impairment; SDLI, Syntactic Developmental Language Impairment; TD, Typically Developing; TLD, Typical Language Development; TDA, Typically Developing Age-Matched Controls; DS, Down Syndrome; FR, French-speaking; HE, Hebrew-speaking.

### Quality assessment

The methodological quality of the included studies was assessed using the Mixed Methods Appraisal Tool (MMAT; Hong et al., 2018). The MMAT was selected because it allows the appraisal of qualitative, quantitative, and mixed-methods studies within a single framework. Each included study was evaluated against the relevant MMAT criteria according to its study design. Quality appraisal was performed independently by both authors, with any discrepancies resolved through discussion until consensus was reached. No studies were excluded on the basis of methodological quality; instead, quality ratings were considered during the interpretation of the findings.

### Data synthesis

Due to substantial heterogeneity in study designs, participant characteristics, languages, assessment tools, and outcome measures, a meta-analysis was not considered appropriate. Therefore, a narrative synthesis was undertaken.

Studies were first grouped according to the structural language domains investigated, including general structural language profiles, phonology, morphology and grammatical marking, syntax and complex sentence processing, lexical-semantic development, narrative and discourse-level language, and the relationship between structural language and broader cognitive processes. Findings within each domain were then compared across studies to identify areas of overlap and differentiation between children with ASD and DLD.

The synthesis focused on patterns of performance across language domains, the severity and consistency of language impairments, and the linguistic and cognitive mechanisms proposed to underlie observed difficulties. Particular attention was paid to similarities and differences between children with ASD and co-occurring language impairment (ASD-LI) and children with DLD.

No data conversions were required. Where studies reported multiple relevant outcomes within a language domain, all relevant findings were extracted and considered in the synthesis. Results were summarized descriptively and are presented according to language domain.

## Results

### Study characteristics and overall pattern of findings

The included studies examined structural language profiles in children with ASD and DLD. Across studies, structural language was assessed using standardized language measures, experimental linguistic tasks, narrative measures, parent-report communication scales, eye-tracking methodologies, electrophysiological paradigms, and intervention-based designs. The reviewed evidence focused primarily on the domains of phonology, morphology, syntax, semantics, and narrative measures reflecting structural language abilities (Table 1). Because the included studies used diverse diagnostic subgroup labels for both ASD (e.g., ASD-LI, ASD-LN, ALI, HFA-LI) and developmental language disorders (e.g., DLD, SLI, LI, ELD, and PLI), references to ASD and DLD in the following narrative synthesis reflect the participant groups examined in the original studies rather than a single binary comparison, with historical language-disorder labels interpreted within the broader DLD framework where appropriate.

Across the included studies, the evidence suggested a pattern of partial overlap alongside important qualitative differences between ASD and DLD. Evidence of overlap was most frequently reported among children with ASD and co-occurring language impairment (ASD-LI), who frequently showed similar performance to children with DLD on standardized language assessments, nonword repetition, sentence repetition, morphosyntactic tasks, and measures of complex syntax. Nevertheless, children with DLD generally exhibited more consistent and pervasive structural language deficits, whereas language performance in ASD was characterized by greater heterogeneity. Differences between the groups were particularly evident in the variability of language profiles and in the influence of broader cognitive, pragmatic, attentional, and processing-related factors on language performance.

### Quality assessment results

The methodological quality of the included studies was generally moderate to high. Most studies employed clearly defined diagnostic criteria, standardized language assessments, and appropriate statistical analyses. Common limitations included relatively small sample sizes, limited consideration of potential confounding variables, and the predominance of cross-sectional designs. Overall, no study was excluded on the basis of methodological quality, and the available evidence was considered sufficient to support synthesis across structural language domains.

### General structural language profiles

Several studies reported overlapping structural language profiles in ASD subgroups with co-occurring language impairment (e.g., ASD-LI, ALI, HFA-LI, LD-ASD) and children with DLD or earlier equivalent language-disorder groups (e.g., SLI, LI, PLI, ELD), although the specific diagnostic subgroups varied across studies. Across receptive and expressive language measures, including sentence formulation, sentence repetition, word structure, vocabulary, and morphosyntax, both groups consistently performed below typically developing (TD) peers and often demonstrated comparable levels of impairment (Brignell et al., 2018; Georgiou and Spanoudis, 2021; Oi et al., 2017; Ramírez-Santana et al., 2019).

Ramírez-Santana et al. (2019) reported substantial overlap between children with SLI and ASD across receptive, expressive, morphosyntactic, and lexical-semantic domains. No significant differences were observed between the clinical groups, whereas both performed significantly below typically developing (TD) peers. In the lexical-semantic domain, children with ASD and SLI demonstrated comparable weaknesses in expressive vocabulary, conceptual tasks, word associations, categorization, and semantic organization, suggesting similar limitations in lexical depth and semantic representation. The authors proposed that both groups experience difficulties establishing semantic classifications, conceptual relationships, and rich word associations, indicating highly similar structural language profiles across multiple language domains.

Similarly, Georgiou and Spanoudis (2021) identified a subgroup of children with ASD and language impairment (ASD-LI) whose structural language profile showed considerable overlap with that of children with DLD. Both ASD-LI and DLD groups performed significantly below TD peers on receptive and expressive language measures, including sentence formulation, sentence repetition, word structure, following directions, and nonword repetition (NWR). Nonword repetition and sentence repetition (SRT) reliably distinguished both clinical groups from TD children but did not consistently differentiate ASD-LI from DLD, suggesting overlap in structural language difficulties. Similar findings were reported by Taylor et al. (2014), who found that children with ASD-LI and SLI performed comparably poorly on nonword repetition tasks, while children with ASD without language impairment performed similarly to TD peers. Together, these findings suggest that repetition-based deficits are primarily associated with co-occurring language impairment rather than ASD itself and support the existence of a shared structural language phenotype across ASD-LI and DLD.

Brignell et al. (2018) reported that children with ASD-LI and DLD exhibited comparable impairments in receptive and expressive language, morphology, and syntax. Across ages 4–7 years, language scores remained consistently below those of TD children, and both groups demonstrated similar developmental trajectories with little evidence of catch-up growth. Structural language outcomes were predicted by early language abilities and nonverbal IQ rather than ASD diagnosis, suggesting that language impairment may represent a dimension that cuts across diagnostic categories.

Using the Japanese Children’s Communication Checklist–2 (CCC-2), Oi et al. (2017) found comparable impairments in peech, syntax, semantics, and coherence in children with ASD and Language Impairment (LI). Both groups scored significantly lower than TD children on all structural language subscales, indicating broad difficulties across multiple language domains. Structural language measures contributed to a common communication factor (GCC), suggesting that impairments in speech, morphosyntax, semantics, and discourse coherence may reflect a shared underlying dimension of communicative ability. Furthermore, structural language performance was unrelated to IQ, indicating that these difficulties could not be explained by general cognitive ability. Although substantial overlap was observed between ASD and LI, children with ASD demonstrated greater difficulties in social interaction and pragmatic communication.

At the same time, several studies identified important differences between ASD subgroups and DLD or earlier equivalent language-disorder groups. Andreou et al. (2025), Doghadze and Gagoshidze (2023), Dockrell et al. (2019), Gibson et al. (2013), and Hare-Harris et al. (2019) reported that although both groups exhibited structural language difficulties, impairments were generally more severe and pervasive in DLD, whereas children with ASD often demonstrated more heterogeneous language profiles.

Andreou et al. (2025) examined structural language abilities in Greek-speaking children with ASD, DLD, and typical development. Significant group differences were found across phonological awareness, morphosyntax, and expressive vocabulary. Children with TD showed the strongest performance, children with DLD the weakest, and children with ASD performed at an intermediate level. The ASD group significantly outperformed the DLD group in phonological awareness and morphosyntax and demonstrated slightly higher expressive vocabulary scores. Overall, the findings suggest overlapping but distinct structural language profiles, with more severe structural language impairments in DLD than in ASD.

Doghadze and Gagoshidze (2023) examined structural and functional language abilities in Georgian-speaking children with ASD, DLD, and typical development. The study identified both overlap and differentiation between ASD and DLD in structural language abilities. Significant group differences were observed across structural and functional language measures. Children with DLD exhibited the highest rates of phonological, semantic, syntactic, and morphological errors, indicating more severe and pervasive structural language impairment. In particular, they produced substantially more phonological errors than both ASD and TD peers, suggesting greater difficulties in speech-sound production. Children with ASD showed fewer phonological difficulties than the DLD group but produced fewer total and different words than both DLD and TD children, while also demonstrating more pronounced pragmatic difficulties. Both ASD and DLD groups exhibited significantly greater morphosyntactic difficulties than TD peers, supporting the presence of shared structural language weaknesses. However, the findings also indicate distinct language profiles, with DLD characterized by more severe structural language deficits and ASD by greater pragmatic impairment.

Dockrell et al. (2019) examined language and literacy outcomes in school-aged children with DLD and ASD. Both groups demonstrated significant weaknesses in receptive vocabulary, grammar, and expressive sentence formulation. However, children with DLD showed more severe impairments across structural language, literacy, and spelling measures than children with ASD. While both groups exhibited difficulties in reading comprehension and spelling, these difficulties were more closely associated with underlying structural language deficits in DLD. In contrast, children with ASD demonstrated greater socio-communicative and pragmatic difficulties. The findings indicate overlapping structural language weaknesses alongside more severe structural deficits in DLD and greater pragmatic difficulties in ASD.

Gibson et al. (2013) compared children with high-functioning autism (HFA), specific language impairment (SLI), and pragmatic language impairment (PLI). Results from the CELF-4 showed that children with SLI and PLI exhibited lower core language scores and marked structural language weaknesses, particularly in expressive language, whereas children with HFA demonstrated relatively stronger language abilities. Both the SLI and PLI groups showed significantly better receptive than expressive language skills, indicating persistent morphosyntactic difficulties, while no receptive-expressive discrepancy was observed in the HFA group. Overall, structural language impairments were most pronounced in SLI, present to a lesser extent in PLI, and relatively preserved in HFA.

Hare-Harris et al. (2019) examined language performance in individuals with ASD, LI, ASD + LI, and unaffected peers using standardized language assessments. Although the ASD + LI and LI-only groups showed similar levels of impairment on conventional language measures, the ASD groups demonstrated significantly greater variability across expressive, syntactic, semantic, metalinguistic, and pragmatic tasks. This increased response dispersion was particularly evident on measures such as Recalling Sentences, Formulated Sentences, Word Classes, and Word Definitions, suggesting a more uneven and atypical language profile in ASD. In contrast, performance on Word Structure did not significantly differentiate ASD and LI groups, indicating similar morphological abilities. Furthermore, greater response variability was associated with higher ASD symptom severity and accurately predicted ASD diagnosis, suggesting that language in ASD is characterized not only by the degree of impairment but also by atypical patterns of language organization and processing.

Overall, the available evidence suggests a pattern of partial overlap in the structural language profiles of ASD subgroups with language impairment and children with DLD or earlier equivalent language-disorder diagnoses, particularly among children with ASD and co-occurring language impairment (ASD-LI). Across receptive and expressive language, morphosyntax, vocabulary, and repetition-based measures, ASD-LI and DLD groups frequently demonstrated comparable levels of impairment and often could not be reliably distinguished using standardized language assessments. These findings support the existence of a shared structural language phenotype across the two disorders. At the same time, important differences emerged. Several studies reported that structural language impairments were generally more severe and pervasive in DLD, whereas language performance in ASD was characterized by greater heterogeneity. Children with ASD more frequently exhibited pragmatic, social-communicative, and discourse-related difficulties, even when structural language abilities were similar to those of children with DLD. Together, the findings suggest that ASD and DLD share significant structural language weaknesses, but differ in the severity, consistency, and broader communicative context of those impairments.

### Phonology and repetition-based markers

Studies examining phonological abilities in ASD subgroups and DLD or earlier equivalent language-disorder groups reported evidence of overlap as well as important differences between the two disorders. Phonological abilities were assessed through nonword repetition, sentence repetition, phonological memory, phonological awareness, speech-sound production, and neurocognitive measures of phonological processing. Several studies reported similar performance between children with ASD-LI and children with DLD on repetition-based tasks, suggesting overlap in phonological memory and language processing (Taylor et al., 2014). At the same time, other studies reported more severe and consistent phonological deficits in DLD, whereas phonological difficulties in ASD appeared more heterogeneous and were often associated with broader cognitive or processing-related factors (Hill et al., 2015; Knowland et al., 2022; Dynia et al., 2019).

Taylor et al. (2014) found that both ASD-LI and SLI groups performed poorly on nonword repetition tasks, while the SLI group showed particularly weak sentence repetition performance. Children with ASD without language impairment performed similarly to TD peers, suggesting that repetition difficulties are primarily associated with co-occurring language impairment rather than ASD itself.

Hill et al. (2015) found that both SLI and ASD with language impairment (ALI) exhibited significant difficulties in nonword repetition and verbal memory compared with ASD children with typical language abilities. Nevertheless, children with SLI performed significantly worse on two - and three-syllable nonword repetition tasks, indicating more severe phonological memory deficits. The authors concluded that although ALI and SLI share repetition-based difficulties, phonological deficits are more pronounced in SLI, suggesting partially overlapping but distinct underlying mechanisms.

Further evidence for qualitative differences was provided by Knowland et al. (2022), who examined neural responses to novel and familiar word forms using an ERP paradigm. Children with DLD showed weaker neural responses and reduced overnight habituation of the N400 component compared to both ASD and TD groups, indicating less efficient consolidation and pre-activation of newly encountered phonological representations. In contrast, children with ASD demonstrated patterns of neural change similar to typically developing peers. The findings suggest that phonological learning and repetition-based consolidation deficits are more pronounced in DLD than in ASD. A lexicality effect (greater N400 negativity for pseudowords than real words) was observed, primarily driven by the ASD group, indicating sensitivity to phonological novelty. However, no robust habituation effects were detected within sessions. Overall, Knowland et al. concluded that lexical encoding and consolidation mechanisms appear atypical in DLD, whereas ASD is characterized by relatively preserved early phonological processing but potentially distinct lexical integration dynamics.

The findings of Doghadze and Gagoshidze (2023) further support the view that phonological difficulties are more severe and pervasive in DLD than in ASD. Children with DLD produced substantially more phonological errors than both ASD and typically developing peers, indicating significant weaknesses in speech-sound production and phonological accuracy. In contrast, although children with ASD demonstrated fewer phonological difficulties, they produced fewer total and different words and exhibited greater pragmatic impairments. This pattern suggests that language difficulties in ASD may not primarily reflect deficits in phonological representation or speech-sound production but rather broader difficulties affecting language use, lexical productivity, and communication. Consequently, while both ASD and DLD are associated with structural language weaknesses, phonological impairment appears to be a more central feature of DLD, whereas language difficulties in ASD are distributed across multiple domains and are frequently accompanied by pragmatic and social-communicative challenges.

Dynia et al. (2019) reported significant phonological awareness deficits in both ASD and language impairment (LI) groups compared with typically developing peers. Children with ASD performed significantly worse than TD children across all phonological awareness measures and, at preschool entry, also showed lower performance than children with LI on several tasks. Although children with ASD demonstrated gains in phonological awareness over the school year comparable to those of TD peers, the performance gap remained, and differences between the ASD and LI groups were no longer significant by the end of the school year. Oral language ability, but not social skills, significantly predicted growth in phonological awareness, highlighting the importance of language skills in phonological development. Overall, both ASD and LI groups exhibited persistent phonological awareness difficulties relative to TD children.

Overall, the evidence suggests partial overlap in phonological abilities between ASD subgroups with language impairment and DLD or earlier equivalent language-disorder groups, particularly on repetition-based tasks. Nevertheless, children with DLD generally exhibited more severe and consistent phonological deficits, whereas phonological difficulties in ASD were characterized by greater heterogeneity and appeared to be influenced by broader cognitive, memory, and processing-related factors.

### Morphology and grammatical marking

Morphological impairment was a consistent feature of DLD and earlier equivalent language-disorder groups (e.g., SLI) and was also frequently observed in ASD subgroups with language impairment (e.g., ASD-LI, ALI, HFA-LI, LD-ASD). Studies examining broad morphosyntactic profiles, tense and aspect marking, clitic production, article use, definiteness marking, pronoun interpretation, and grammatical judgment tasks consistently reported that children with ASD-LI and DLD performed below typically developing peers and often did not differ significantly from one another (Chen et al., 2022; Durrleman and Delage, 2016; Ellis Weismer et al., 2017; Huang and Finestack, 2020; Prévost et al., 2018; Schaeffer et al., 2017; Zhang et al., 2024). Across languages, evidence of overlap was reported in grammatical morphology, although several studies identified differences in error patterns and underlying mechanisms.

Huang and Finestack (2020) provided a detailed comparison of morphosyntactic abilities in children with DLD and language disorder associated with ASD (LD-ASD). Using both a standardized grammar assessment (SPELT-3) and language sample analysis (IPSyn), they reported highly similar morphosyntactic profiles across groups, with no significant overall differences. Only a small number of structures differentiated the groups: children with DLD performed better on participles, the conjunction “and,” and infinitives, whereas children with LD-ASD performed better on several question-related structures. Overall, the vast majority of grammatical structures showed similar performance, suggesting considerable overlap in morphosyntactic impairment between DLD and LD-ASD. The authors concluded that morphosyntactic difficulties in LD-ASD largely resemble those observed in DLD, although subtle differences may emerge depending on the assessment method used.

Chen et al. (2022) examined the comprehension and production of Mandarin aspect markers (*zai-*, *−le*, *−zhe*, *−guo*) in children with HFA-LI, DLD, and TD peers. Both HFA-LI and DLD groups performed significantly worse than TD children in the comprehension of *zai-*, *−le*, and *-guo*, while no group differences were found for *-zhe*. No significant differences emerged between the HFA-LI and DLD groups, indicating highly similar comprehension profiles. In production, both clinical groups produced significantly fewer target aspect markers and more bare verbs, irrelevant responses, and alternative aspect markers, particularly for *-guo*. More than half of the children in both groups failed to produce any correct instances of *-guo*. Although lexical aspect influenced performance in all groups, children with HFA-LI and DLD showed less accurate use of aspect-verb combinations. Overall, the findings indicate similar deficits in grammatical morphology (aspect marking) in HFA-LI and DLD, suggesting overlap in their structural language profiles.

Prévost et al. (2018) examined the production of nominative, reflexive, and accusative clitics in French-speaking children with ASD and SLI. Children with ASD and structural language impairment (ASD-LI) showed production patterns highly similar to those of children with SLI. In both groups, third-person accusative clitics were produced significantly less accurately than other clitic types, confirming their role as a marker of structural language impairment. No significant differences were found between ASD-LI and SLI, and error patterns were largely characterized by omissions and substitutions. Performance was unrelated to nonverbal IQ in the ASD group. Overall, the findings suggest that clitic production difficulties in ASD-LI are structural in nature and closely resemble those observed in SLI.

Durrleman and Delage (2016) similarly investigated third-person accusative clitics, a well-established clinical marker of SLI. Both ASD and SLI groups performed significantly below age- and language-matched TD controls and did not differ significantly from one another, indicating overlap in complex morphosyntactic production. Both groups also showed comparable difficulties in expressive morphosyntax. However, ASD was more heterogeneous, with approximately one-third of participants demonstrating intact grammatical abilities. A notable difference emerged for first-person accusative clitics: children with ASD performed significantly worse than both SLI and TD groups, a finding attributed to Theory of Mind and perspective-taking demands rather than purely syntactic deficits. Working memory was associated with clitic production in both clinical groups, whereas nonverbal reasoning was not. Overall, the findings support a pattern of overlap between ASD and SLI in morphosyntax, while highlighting greater heterogeneity and additional pragmatic-cognitive influences in ASD.

Schaeffer et al. (2017) examined article choice and definiteness marking in children with HFA, SLI, and TD peers. Both HFA and SLI groups performed significantly below TD children in definite contexts and frequently overgenerated indefinite articles in place of definite articles. No significant differences were found between the HFA and SLI groups. Both clinical groups also showed reduced phonological memory and verbal working memory. The findings suggest overlap between HFA and SLI in definiteness marking and indicate that these difficulties may be linked more closely to memory limitations than to Theory of Mind deficits.

Zhang et al. (2024) investigated the comprehension of definite noun phrases and pronouns in children with DLD, autism with language impairment (ALI), autism with normal language (ALN), and TD peers. Both the DLD and ALI groups performed significantly below TD and ALN groups on measures of definiteness and pronoun interpretation, with no significant differences between the two language-impaired groups on most measures. However, children with ALI showed significantly poorer comprehension of third-person pronouns than children with DLD, suggesting additional ASD-related processing difficulties. The findings indicate overlap in definiteness processing deficits while highlighting potential differences in referential processing.

Schaeffer (2018) reported a different pattern. Children with SLI demonstrated significant weaknesses in several morphosyntactic domains, including the mass-count distinction, subject-verb agreement, and sentence repetition. In contrast, children with HFA performed at levels comparable to TD peers and significantly outperformed the SLI group on these measures. Grammatical performance in the SLI group was associated with both verbal and nonverbal working memory. These findings suggest that morphosyntactic impairment is a core feature of SLI, whereas structural language abilities may be relatively preserved in some children with HFA.

Ellis Weismer et al. (2017) examined morphosyntactic processing using a grammatical judgment task targeting tense and auxiliary omissions. Children with SLI demonstrated significantly lower sensitivity to grammatical violations than TD peers, confirming persistent morphosyntactic weaknesses. Children with ASD showed intermediate performance, reflecting the heterogeneity of language abilities within the autism spectrum. When participants were regrouped according to language status rather than diagnosis, children with language impairment (SLI and ASD-LI) performed significantly worse than children with normal language abilities, suggesting that morphosyntactic performance was more strongly associated with language impairment than with diagnostic category. Nonverbal working memory also significantly predicted grammatical processing performance.

Overall, the evidence suggests overlap in grammatical morphology between ASD subgroups with language impairment and DLD or earlier equivalent language-disorder groups. Similar difficulties were observed across aspect marking, clitic production, article choice, definiteness marking, pronoun interpretation, tense morphology, and broader morphosyntactic measures. Several studies reported little or no difference between ASD-LI and DLD on standardized grammatical assessments, suggesting a partially overlapping morphosyntactic phenotype. Nevertheless, important qualitative differences emerged. Whereas errors in DLD were typically grammatical in nature, children with ASD more frequently demonstrated heterogeneity, greater response variability, and difficulties influenced by discourse integration, referential processing, perspective-taking, attention, or working memory. These findings suggest that similar morphosyntactic performance may reflect partially distinct underlying mechanisms in the two disorders.

### Syntax and complex sentence processing

Syntax was the most extensively examined structural language domain across the reviewed studies. Research focused on a wide range of complex constructions, including wh-questions, relative clauses, passive sentences, clitic pronouns, reflexives, sentential complements, control structures, noncanonical word order, and other movement-derived dependencies. Across studies, children with ASD accompanied by language impairment (ASD-LI/ALI) and children with DLD frequently reported similar difficulties in the comprehension and production of complex syntactic structures, particularly when processing demands increased. Nevertheless, detailed analyses often revealed important qualitative differences, suggesting that superficially similar performance may arise from distinct underlying mechanisms.

Several studies suggested overlap between ASD subgroups with language impairment and DLD or earlier equivalent language-disorder groups in syntactic processing. Prévost et al. (2017) found that children with ASD and SLI showed remarkably similar sensitivity to morphosyntactic complexity in both the production and comprehension of French wh-questions. Both groups preferred simpler wh-in-situ structures and avoided more complex constructions involving movement and inversion, indicating similar difficulties with syntactic computation. Complexity effects were evident across modalities, with more complex wh-questions generating lower accuracy in both groups. Nonverbal ability was not significantly associated with syntactic performance, suggesting that morphosyntactic difficulties were independent of general cognitive functioning. Although children with ASD produced fewer appropriate questions overall, the authors attributed this mainly to pragmatic task demands rather than greater structural language impairment.

Fortunato-Tavares et al. (2015) similarly reported overlapping syntactic difficulties in children with HFA and SLI. Both groups performed significantly below TD peers on predicate attachment and reflexive binding tasks and showed difficulties establishing hierarchical syntactic relations. Under increased working-memory demands, children with SLI demonstrated reduced accuracy and more syntactic errors, reaching performance levels comparable to those observed in HFA. The findings suggest overlap in sentence-comprehension difficulties while highlighting the influence of working memory on syntactic processing.

Moreno-Pérez et al. (2020) examined reflexive and clitic pronoun comprehension in children and adolescents with ASD-HL, ASD-LL, DLD, and TD controls using both online eye-tracking and offline comprehension tasks. No significant group differences were observed in reflexive or clitic pronoun comprehension. Eye-tracking data revealed similar processing patterns across ASD, DLD, and control groups, while offline accuracy scores showed no significant differences between ASD-LL, DLD, and language-matched controls. The authors concluded that reflexive and clitic pronoun comprehension do not provide reliable linguistic markers for differentiating ASD from DLD.

Additional evidence for overlap was reported by Garrido et al. (2024), who examined receptive grammar in children with ALI, ALN, DLD, and TD peers. Performance on simple grammatical structures was comparable across groups once children reached a receptive vocabulary age equivalent of approximately 7 years and 8 months. However, both ALI and DLD groups performed significantly below ALN and TD children on more complex non-canonical and reversible constructions. The findings suggest that syntactic complexity represents a shared area of difficulty for both disorders, although subtle differences in performance patterns indicate that the nature of the impairment may not be identical.

Dai et al. (2025) investigated the effects of syntactic complexity on the comprehension of Mandarin passive clauses and wh-questions in children with DLD, ALI, and TD peers. Both clinical groups performed significantly below TD children and demonstrated highly similar patterns of non-target responses. Both groups showed a short-long passive asymmetry, performing better on short passives than on syntactically more complex long passives, suggesting that movement and intervention effects negatively influenced comprehension. Likewise, both groups experienced difficulties with wh-questions involving complex syntactic dependencies. However, a who-which asymmetry emerged only in the ALI group, indicating additional difficulties related to referentiality and discourse processing. Overall, the findings suggest overlap in complex syntactic processing alongside subtle ASD-specific vulnerabilities.

Intervention studies further supported similarities between ASD and DLD in syntactic development. Azevedo et al. (2025) investigated the effects of the PROsyntax intervention on expressive and receptive syntax in preschool children with ASD and DLD. Children in both groups demonstrated significant improvements following intervention, with broadly similar patterns of responsiveness. Although children with DLD achieved higher post-intervention scores, children with ASD demonstrated proportionally larger gains, particularly in expressive syntax. The absence of significant group-by-intervention interactions suggests comparable responsiveness to explicit syntactic intervention across disorders.

Similarly, Durrleman et al. (2019) examined whether training sentential complement clauses could improve grammatical abilities and Theory of Mind in children with ASD and DLD. Children receiving syntactic training showed significant improvements in complement-clause comprehension and false-belief reasoning, whereas lexical training produced no comparable gains. Children with ASD and DLD demonstrated similar patterns of improvement, and gains in complement-clause mastery significantly mediated improvements in Theory of Mind performance.

Related findings were reported by Durrleman et al. (2017), who examined the relationship between complement-clause comprehension and Theory of Mind in children with SLI, ASD, and TD peers. Both clinical groups demonstrated similar levels of performance on complement-clause tasks and Theory of Mind measures. Complement clauses involving communication verbs were particularly strong predictors of false-belief understanding. Children matched on complement-clause abilities also demonstrated comparable Theory of Mind performance regardless of diagnostic group, highlighting the central role of complex syntax in social-cognitive development.

Despite these similarities, several studies reported important qualitative dissociations between ASD and DLD. Sukenik and Friedmann (2018) found that children with ASD and SyDLI achieved similar overall accuracy on relative clauses, wh-questions, topicalization, and sentence repetition tasks, yet demonstrated markedly different language profiles. Children with SyDLI showed a selective morphosyntactic deficit characterized by pronounced difficulties with movement-derived structures such as object relatives. In contrast, children with ASD exhibited greater heterogeneity, with some performing at ceiling and others showing broad impairments across both simple and complex sentence types. Error analyses revealed that SyDLI participants primarily produced syntactic errors, whereas ASD participants frequently produced pragmatically inappropriate and discourse-related responses.

Sukenik et al. (2021) reported a similar pattern in wh-question production. Although ASD and DLD groups showed similarly low rates of correct *wh*-question production and performed significantly below TD children, detailed error analyses revealed distinct profiles. Children with DLD primarily produced ungrammatical syntactic errors, whereas children with ASD frequently produced pragmatically inappropriate, perseverative, and contextually irrelevant responses. These findings suggest that similar surface-level performance conceals different underlying mechanisms, with DLD reflecting core morphosyntactic impairment and ASD involving greater pragmatic and discourse-level difficulties.

Nie et al. (2025) compared the comprehension and production of Mandarin ba and bei constructions in preschool children with DLD, ALI, and TD peers. Both clinical groups performed significantly below TD children on comprehension and production tasks, and no significant differences in overall accuracy were observed between DLD and ALI. However, qualitative analyses revealed important differences. Children with DLD primarily produced syntactic and grammatical errors, whereas children with ALI frequently produced pragmatically inappropriate and contextually irrelevant responses. Furthermore, nonverbal working memory was associated with performance in the DLD group but not in ALI. The authors concluded that similar levels of performance masked distinct linguistic and cognitive mechanisms.

Issa et al. (2023) examined comprehension of who - and which-wh questions in Jordanian Arabic-speaking children with ASD, SDLI, and TD peers. Both ASD and SDLI groups performed significantly below TD children and showed greater difficulty with object questions than subject questions. Which-questions were consistently more difficult than who-questions, particularly object which-questions. Although both groups demonstrated syntactic comprehension deficits, children with SDLI achieved higher overall accuracy than children with ASD, suggesting more severe difficulties in the ASD group.

Overall, the evidence suggests overlap in complex syntax between ASD subgroups with language impairment and DLD or earlier equivalent language-disorder groups. Both groups consistently experience difficulties with movement-derived constructions, noncanonical word order, passives, relative clauses, wh-questions, and sentential complements, and they often achieve comparable levels of accuracy on standardized and experimental syntactic tasks. However, detailed error analyses reveal important differences. Children with DLD typically exhibit selective morphosyntactic impairments characterized by structurally constrained errors, whereas children with ASD frequently demonstrate greater heterogeneity, broader performance variability, and increased pragmatic, discourse-related, or referential processing difficulties. These findings suggest that similar surface-level syntactic performance may reflect partially distinct linguistic and cognitive mechanisms in the two disorders.

### Semantics, vocabulary, and lexical processing

Findings in the lexical-semantic domain suggested partial overlap between ASD subgroups with language impairment and DLD or earlier equivalent language-disorder groups, particularly in vocabulary knowledge, semantic organization, word learning, and lexical processing. Several studies reported similar performance across clinical groups on measures of expressive vocabulary, conceptual knowledge, semantic categorization, word associations, lexical decision, and novel word learning (Gladfelter et al., 2019; Haebig et al., 2015; Ricketts et al., 2015). However, other studies identified important differences in lexical learning mechanisms, semantic processing, and figurative language comprehension, suggesting that superficially similar performance may reflect distinct underlying cognitive processes.

Haebig et al. (2015) examined lexical-semantic organization using a lexical decision task. Children with ASD and SLI demonstrated lexical processing performance similar to vocabulary-matched typically developing peers, suggesting broadly similar lexical-semantic organization across groups. All groups were more accurate when processing words from larger semantic networks than words from smaller semantic networks. However, follow-up analyses revealed a weaker semantic network effect in the SLI group, indicating that semantic representations may be less densely connected or less efficiently organized. In contrast, children with ASD showed semantic network facilitation patterns similar to typically developing peers. No significant differences were found between ASD and SLI in overall lexical decision accuracy or reaction time. Executive-function abilities, particularly updating and shifting, significantly predicted lexical-semantic processing across groups, suggesting common cognitive mechanisms underlying lexical access.

Ricketts et al. (2015) investigated spoken vocabulary learning using a nonword-referent learning task. Children with ASD, SLI, and typical development all demonstrated significant learning over time, indicating successful acquisition of phonological-semantic associations. Written word forms significantly enhanced vocabulary learning across groups, and no differences were found in learning accuracy. However, children with ASD showed slower access to newly learned words, suggesting reduced processing efficiency, whereas children with SLI demonstrated weaker baseline vocabulary and broader structural language abilities despite similar experimental learning performance. These findings suggest shared vocabulary-learning mechanisms alongside disorder-specific differences in processing speed and broader language outcomes.

Gladfelter et al. (2019) examined how children with ASD, DLD, and typical language development use visual and verbal information to construct semantic representations of newly learned words. Both ASD and DLD groups produced significantly more visually presented semantic features than typically developing children, suggesting a greater reliance on visual information during lexical learning. No significant differences were observed between ASD and DLD, indicating similar lexical-semantic learning profiles. Furthermore, all groups increased their use of semantic features presented through combined visual-verbal input across learning sessions, demonstrating the benefits of multimodal learning. The findings suggest that children with ASD and DLD can acquire new semantic information effectively but may rely more heavily on visual support than typically developing peers.

Although several studies demonstrated overlap, others identified important differences in lexical learning and semantic processing. Haebig et al. (2017) investigated statistical word learning and vocabulary acquisition in children with ASD, SLI, and typical development. Children with SLI performed significantly worse than both ASD and TD groups on statistical learning and fast-mapping tasks, indicating difficulties acquiring and encoding new lexical items. In contrast, children with ASD showed intact statistical learning abilities and performed similarly to TD peers. Further analyses revealed that children with ASD and language impairment exhibited subtle weaknesses in fast mapping, whereas ASD children with normal language abilities demonstrated age-appropriate performance. The findings suggest that lexical-semantic difficulties in SLI are associated with deficits in statistical learning and lexical acquisition, whereas vocabulary learning mechanisms are relatively preserved in ASD, particularly in the absence of language impairment.

Walenski and Love (2017) examined real-time idiom comprehension using a cross-modal lexical priming paradigm. Children with SLI demonstrated activation of idiomatic meanings similar to typically developing peers, indicating relatively preserved semantic representations of figurative language. In contrast, children with ASD showed significant activation of literal meanings but not idiomatic meanings, suggesting a literal-meaning bias and reduced access to figurative interpretations. The authors interpreted these findings as evidence of atypical lexical-semantic processing and semantic integration in ASD, whereas children with SLI exhibited relatively intact semantic mechanisms for idiom comprehension.

Knowland et al. (2022) investigated lexical-semantic processing and word learning using the N400 ERP component. Children with DLD demonstrated weaker neural responses and reduced changes in lexical-semantic activation across sessions, suggesting less efficient lexical representation, weaker semantic network activation, and reduced consolidation of newly learned words. In contrast, children with ASD showed lexical-semantic processing patterns largely comparable to those of typically developing peers, although they demonstrated stronger differentiation between words and pseudowords. The findings indicate reduced lexical-semantic consolidation in DLD but relatively preserved semantic processing in ASD.

Overall, the lexical-semantic findings suggest partial overlap between ASD and DLD. The included studies frequently reported weaknesses in vocabulary knowledge, semantic organization, lexical depth, and word learning in both groups. However, important differences emerged in the mechanisms underlying lexical processing. Children with DLD more consistently exhibited deficits in statistical learning, lexical consolidation, and semantic network organization, whereas lexical-semantic performance in ASD was more heterogeneous and strongly influenced by the presence or absence of co-occurring language impairment. Furthermore, ASD was associated with distinctive difficulties in figurative language and idiom comprehension, reflecting broader differences in semantic integration and contextual processing. These findings suggest that although ASD and DLD may display similar levels of lexical-semantic performance on some tasks, the underlying nature of their semantic difficulties is not always equivalent.

### Discourse and narrative measures of structural language

Narrative studies provided additional evidence regarding structural language abilities in ASD subgroups and DLD or earlier equivalent language-disorder groups. Measures of narrative microstructure, including mean length of utterance, syntactic complexity, lexical diversity, and semantic richness, frequently reported overlapping weaknesses in both groups. In contrast, narrative macrostructure and discourse-level measures often highlighted greater difficulties in ASD, particularly in aspects of narrative production requiring social-cognitive and pragmatic processing.

Norbury et al. (2014) compared narrative production in children with ASD, LI and TD peers. Although children with ASD had age-appropriate scores on standardized language tests, both ASD and LI groups produced narratives that were structurally weaker than those of TD children. Specifically, they used fewer different words, produced shorter utterances, demonstrated lower semantic diversity, and showed poorer narrative macrostructure. No significant differences were found between ASD and LI groups on lexical diversity, utterance length, syntactic complexity, or semantic richness, indicating substantial overlap in structural language difficulties during narrative production. Children with LI showed the lowest use of internal-state language and produced significantly fewer semantically relevant story propositions than TD peers, while the ASD group demonstrated intermediate performance. Overall, the findings suggest that ASD and LI exhibit similar weaknesses in lexical-semantic and syntactic aspects of narrative discourse despite differing clinical profiles.

Govindarajan and Paradis (2022) examined narrative macrostructure and microstructure in bilingual children with ASD, DLD, and TD peers. Narrative production was elicited using the Edmonton Narrative Norms Instrument (ENNI), and analyses included measures of story grammar, syntactic complexity, mean length of utterance, lexical diversity, and story length. At the microstructural level, both ASD and DLD groups demonstrated reduced syntactic complexity compared with TD children, and no significant differences were found between the clinical groups in syntactic complexity or utterance length, indicating overlap in structural language performance. Lexical diversity and story length also did not differ significantly across groups. At the macrostructural level, both ASD and DLD groups demonstrated weaker narrative organization than TD peers and performed similarly on overall story grammar measures. However, children with ASD produced fewer internal reactions than children with DLD, suggesting greater difficulty with perspective-taking and mental-state representation. Thus, while ASD and DLD exhibited similar structural language weaknesses, ASD was additionally characterized by difficulties in narrative elements requiring social-cognitive processing.

Nielsen et al. (2025) compared narrative production in children with ASD, DLD, and TD peers using narratives elicited from social and physical animations. Both ASD and DLD groups included significantly fewer relevant content elements than TD children, indicating weaknesses in narrative macrostructure and content organization. Children with ASD also produced shorter narratives than TD peers. When animations were presented in smaller segments, ASD children generated more relevant information, suggesting that memory and organizational demands influenced narrative performance. Although intentionality attribution did not differ significantly between ASD and TD children, both clinical groups demonstrated reduced narrative content relative to TD peers. These findings indicate that narrative difficulties in ASD and DLD involve challenges in organizing and integrating information across discourse, in addition to sentence-level language weaknesses.

Overall, narrative studies suggested overlap between ASD and DLD in measures of narrative microstructure. Both groups frequently produced shorter utterances, less complex syntax, reduced lexical diversity, and weaker semantic content than typically developing peers, with few consistent differences between ASD and DLD. However, greater differentiation emerged at the discourse level. Children with ASD more often demonstrated difficulties with referential cohesion, character introduction, outcomes, internal reactions, mental-state language, extraneous utterances, and other narrative elements requiring social-cognitive and pragmatic processing. In contrast, narrative difficulties in DLD appeared more closely related to underlying structural language weaknesses. These findings suggest that narrative microstructure primarily reflects overlapping structural language vulnerabilities, whereas narrative macrostructure and discourse-level measures may better differentiate ASD from DLD.

### Pragmatics–structure Interface and processing factors

Several studies suggested that similarities between ASD subgroups with language impairment and DLD or earlier equivalent language-disorder groups become less pronounced when tasks require the integration of structural language with pragmatics, discourse context, prosody, Theory of Mind, or other cognitive processes. Although children with ASD and DLD often demonstrated comparable performance on measures of structural language, important differences emerged when successful task performance depended on the coordination of linguistic and non-linguistic information.

Andrés-Roqueta and Katsos (2020) examined the contribution of structural language and Theory of Mind (ToM) to pragmatic abilities in children with ASD and DLD. On linguistic-pragmatic tasks primarily supported by structural language, children with ASD and DLD performed similarly, and structural language was the only significant predictor of performance. In contrast, on social-pragmatic tasks requiring both language and mental-state reasoning, children with ASD performed significantly worse than children with DLD despite comparable structural language abilities. Both structural language and ToM contributed to performance, suggesting that pragmatic difficulties in ASD extend beyond language impairment alone.

Similarly, Eberhardt and Nadig (2016) demonstrated that structural language ability, rather than diagnostic status, was the primary predictor of context-sensitive language comprehension. Children with ASD and weaker language abilities, as well as children with LI, showed reduced use of contextual information when interpreting ambiguous homonyms and produced fewer context-appropriate interpretations. In contrast, children with ASD who had intact structural language skills performed similarly to typically developing peers. Across analyses, structural language measures were stronger predictors of performance than ASD diagnosis or nonverbal IQ, indicating overlap between ASD with language impairment and LI in context-sensitive language processing.

Likewise, Huang et al. (2025) investigated the interpretation of non-interrogative Mandarin wh-words in children with autism and language impairment (ALI) and DLD. Both groups demonstrated similar difficulties in grammatical statement conditions requiring integration of syntactic and semantic information, indicating shared weaknesses in structural language processing. However, children with ALI showed additional difficulties using prosodic cues to distinguish statements from questions and displayed stronger associations between language performance and cognitive measures. These findings suggest that although ALI and DLD may show similar levels of structural language performance, they differ in the cognitive and linguistic mechanisms supporting language processing.

Overall, these studies suggest that similar performance on structural language tasks does not necessarily reflect identical underlying language profiles. Whereas difficulties in DLD are more consistently linked to core structural language weaknesses, language difficulties in ASD often appear to be influenced by additional pragmatic, discourse-level, prosodic, and social-cognitive factors. Consequently, overlap between ASD and DLD is most apparent on measures of structural language, while differences become increasingly evident when language must be integrated with broader communicative and cognitive processes.

### Summary of results

Across the included studies, the evidence suggested a pattern of partial overlap between ASD subgroups with co-occurring language impairment (e.g., ASD-LI, ALI, HFA-LI, LD-ASD) and children with DLD or earlier equivalent language-disorder groups (e.g., SLI, LI, PLI, ELD) in structural language abilities. Evidence of overlap was reported across phonology, morphology, syntax, semantics, and narrative microstructure. The included studies frequently reported similar performance between children with ASD-LI and DLD on standardized language assessments, nonword repetition, sentence repetition, grammatical morphology, complex syntax, vocabulary measures, and narrative language tasks. Taken together, these findings suggest that ASD subgroups with co-occurring language impairment and children with DLD share several structural language characteristics across multiple domains.

Despite these similarities, important differences emerged across domains. Children with DLD generally demonstrated more consistent and pervasive structural language impairments, whereas language performance in ASD was characterized by greater heterogeneity. Several studies identified subgroups of children with ASD whose language abilities were comparable to those of typically developing peers, while others exhibited profiles similar to DLD. Qualitative analyses further revealed distinct error patterns. Children with DLD tended to produce structurally constrained grammatical and morphosyntactic errors, whereas children with ASD more frequently demonstrated pragmatic, discourse-related, referential, or contextually inappropriate responses. Differences were particularly evident in tasks requiring integration of structural language with discourse, prosody, Theory of Mind, or contextual information.

Overall, the reviewed studies frequently reported overlapping structural language characteristics between ASD subgroups with co-occurring language impairment and children with DLD or earlier equivalent language-disorder diagnoses. However, they also identified important differences in the consistency, heterogeneity, and qualitative nature of language difficulties across the two conditions. While DLD appears to be characterized primarily by core structural language deficits, language difficulties in ASD are often influenced by additional social-cognitive, pragmatic, and processing-related factors. These findings are consistent with a model of partial overlap rather than complete equivalence between the language phenotypes associated with ASD and DLD.

**Figure 2:**
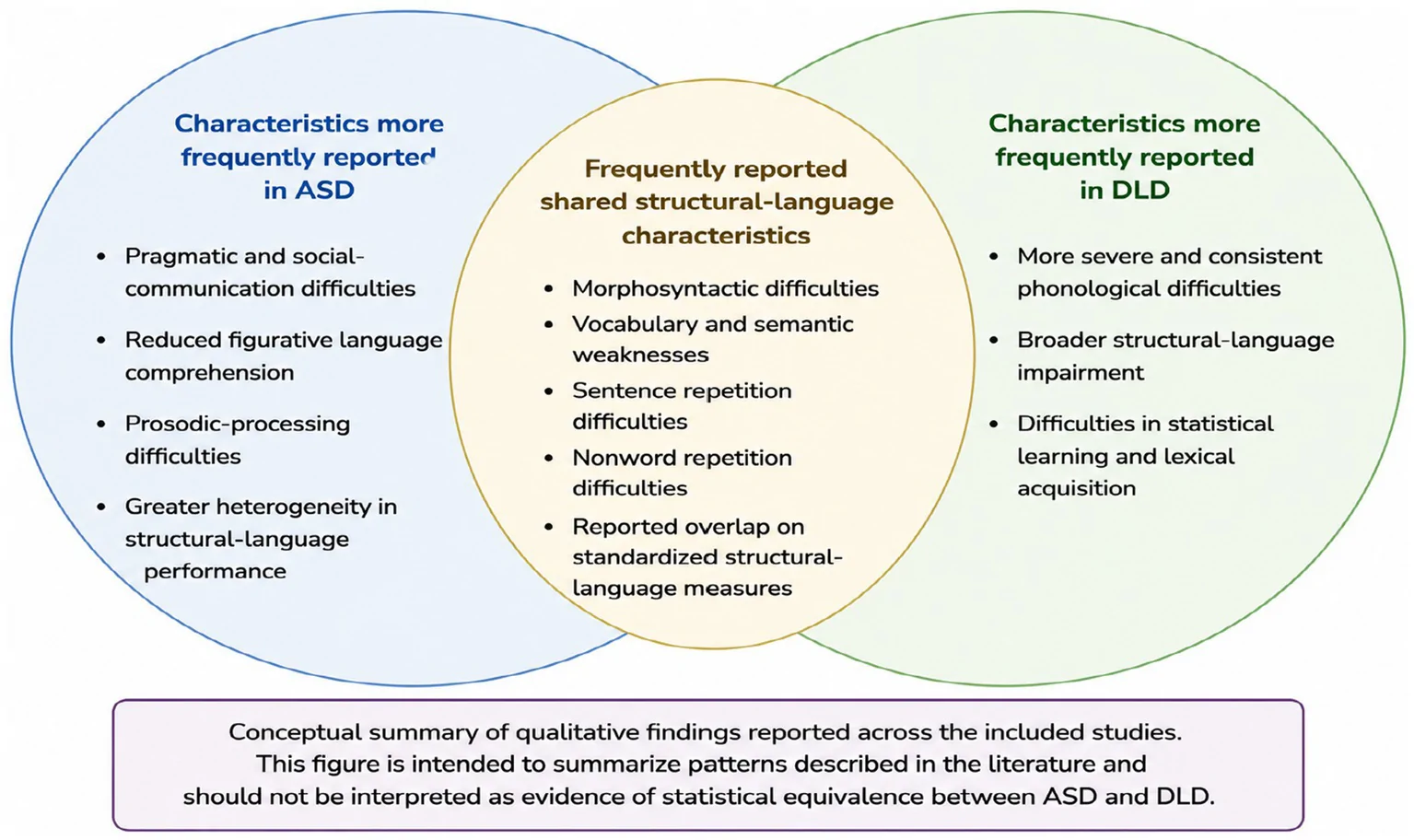
Conceptual synthesis of reported structural language characteristics in autism spectrum disorder (ASD) and developmental language disorder (DLD). The figure summarizes structural language characteristics reported more frequently in ASD, reported more frequently in DLD, and commonly reported across both conditions in the included studies. It represents a qualitative conceptual synthesis of the evidence and is intended to complement the narrative review rather than provide quantitative estimates of effect size or statistical equivalence.

To facilitate interpretation of the narrative synthesis, Figure 2 provides a conceptual overview of the structural language characteristics reported more frequently in ASD, reported more frequently in DLD, and commonly reported across both conditions. The figure represents a qualitative synthesis of the included studies and is intended to summarize reported patterns in the literature rather than provide a quantitative estimate of effect size or statistical equivalence.

## Discussion

This systematic review synthesized findings from 44 studies examining structural language abilities in children with Autism Spectrum Disorder (ASD) and Developmental Language Disorder (DLD). Across phonological, morphological, syntactic, lexical-semantic, and narrative domains, the evidence suggested a pattern of partial overlap between the two conditions, particularly among children with ASD and co-occurring language impairment (ASD-LI). However, important qualitative differences also emerged, suggesting that similar linguistic and behavioral outcomes may arise from distinct underlying mechanisms. Overall, the findings are consistent with a model of partial phenotypic overlap, indicating that ASD-LI and DLD share several structural language characteristics but do not represent a single, unified language disorder.

### Overall patterns of similarity and difference

The findings of the present review suggest overlap in the structural language profiles of children with DLD and children with ASD accompanied by language impairment (ASD-LI). Across receptive and expressive language, vocabulary, morphosyntax, and repetition-based measures, ASD-LI and DLD groups frequently showed similar performance and often did not differ significantly on standardized language assessments (Brignell et al., 2018; Georgiou and Spanoudis, 2021; Oi et al., 2017; Ramírez-Santana et al., 2019; Taylor et al., 2014). These findings are consistent with previous suggestions that language impairment in a subgroup of ASD children closely resembles the structural language difficulties traditionally associated with DLD and may represent a shared language phenotype that transcends diagnostic categories (Bishop, 2010a,b; Kjelgaard and Tager-Flusberg, 2001; Tager-Flusberg and Joseph, 2003).

A particularly consistent finding across studies was that structural language performance was more strongly associated with language impairment status than with diagnostic classification. Children with ASD-LI frequently showed profiles similar to those of children with DLD across sentence formulation, sentence repetition, word structure, vocabulary, and morphosyntactic measures, whereas ASD children without language impairment generally performed similarly to TD peers (Georgiou and Spanoudis, 2021; Taylor et al., 2014). Furthermore, structural language outcomes were often related to factors such as early language ability, working memory, and nonverbal cognitive skills rather than ASD diagnosis itself (Brignell et al., 2018; Oi et al., 2017). Together, these findings support dimensional approaches to neurodevelopmental disorders that emphasize language ability as an important source of variation across diagnostic groups.

Despite these similarities, the review also identified important distinctions between ASD and DLD. Several studies reported that structural language impairments were generally more severe and pervasive in DLD, particularly in phonology, morphosyntax, vocabulary, literacy, and expressive language skills (Andreou et al., 2025; Dockrell et al., 2019; Doghadze and Gagoshidze, 2023; Gibson et al., 2013). In contrast, language performance in ASD was characterized by greater heterogeneity, with some children demonstrating language abilities comparable to those of TD peers and others exhibiting impairments similar to those observed in DLD (Gibson et al., 2013; Hare-Harris et al., 2019). This variability is consistent with contemporary models describing multiple language phenotypes within the autism spectrum (Silleresi, 2023; Vogindroukas et al., 2022).

Another recurring finding concerned the broader communicative context of structural language difficulties. While children with ASD-LI and DLD often achieved similar scores on formal language measures, children with ASD more frequently demonstrated pragmatic, social-communicative, and discourse-level difficulties that extended beyond core structural language domains (Doghadze and Gagoshidze, 2023; Dockrell et al., 2019; Oi et al., 2017). Increased response variability and atypical patterns of language organization observed in ASD further suggest that similar levels of structural language performance may arise from partially different underlying cognitive and linguistic mechanisms (Hare-Harris et al., 2019). Consequently, similar scores on standardized assessments should not necessarily be interpreted as evidence of identical language profiles.

Overall, the evidence suggests that ASD-LI and DLD share several structural language characteristics and are consistent with a model of partially overlapping language phenotypes. However, the greater severity and consistency of structural language impairments typically reported in DLD, together with the increased heterogeneity and pragmatic difficulties characteristic of ASD, suggest that the relationship between the two disorders is one of partial overlap rather than equivalence. Future research should continue to investigate both shared and disorder-specific mechanisms using longitudinal designs, detailed error analyses, and experimental methodologies capable of capturing qualitative differences that may not be be apparent on standardized language measures alone.

### Phonological development and repetition-based markers

The findings of the present review suggest both overlap and differentiation in the phonological profiles of children with ASD-LI and DLD. Across studies, repetition-based measures such as nonword repetition and sentence repetition consistently identified weaknesses in both groups, supporting the view that phonological memory and language-processing difficulties are shared characteristics of language impairment regardless of diagnostic category (Hill et al., 2015; Taylor et al., 2014). In particular, children with ASD-LI frequently demonstrated performance comparable to that of children with DLD on nonword repetition tasks, whereas ASD children without language impairment often performed similarly to TD peers (Taylor et al., 2014). These findings are consistent with the notion that phonological difficulties in ASD are largely associated with co-occurring language impairment rather than autism itself. These findings are consistent with previous proposals that repetition-based tasks may function as clinical markers of language impairment across diagnostic categories (Bishop and Snowling, 2004; Graf Estes et al., 2007).

Despite this overlap, several studies indicated that phonological impairments tend to be more severe and consistent in DLD than in ASD. Children with DLD showed greater difficulties in phonological memory, speech-sound production, and repetition-based tasks, suggesting a more pervasive phonological deficit (Hill et al., 2015). Similarly, evidence from Georgian-speaking children demonstrated that phonological errors were considerably more frequent in DLD than in ASD, although both clinical groups exhibited structural language difficulties relative to TD peers (Doghadze and Gagoshidze, 2023). These findings are consistent with broader evidence suggesting that phonological impairment represents a core feature of DLD, whereas phonological difficulties in ASD are more variable and often less pronounced.

The review also highlights important differences in the underlying mechanisms associated with phonological performance. Neurocognitive evidence suggests that children with DLD experience difficulties in the encoding, consolidation, and activation of phonological and lexical representations, reflected in weaker neural responses and reduced consolidation of newly learned word forms (Knowland et al., 2022). In contrast, children with ASD demonstrated neural responses that were more similar to those of TD peers and showed sensitivity to phonological novelty, suggesting relatively preserved early phonological processing despite broader language difficulties (Knowland et al., 2022). Likewise, phonological awareness studies indicate that although children with ASD and language impairment both experience difficulties relative to typically developing peers, growth in phonological awareness is more strongly associated with oral language abilities than with autism-related social characteristics (Dynia et al., 2019).

Taken together, these findings suggest that phonological difficulties constitute an important area of overlap between ASD-LI and DLD, particularly on repetition-based measures that tap phonological memory and language processing. However, the greater severity, consistency, and neurocognitive atypicalities reported in DLD suggest that phonological impairment is more central to the DLD phenotype. In contrast, phonological difficulties in ASD appear to reflect a more heterogeneous profile that is influenced by language level, memory processes, and broader cognitive factors. Future research should combine behavioral and neurocognitive methodologies to clarify the mechanisms underlying phonological development and to determine whether specific phonological markers can reliably distinguish ASD-LI from DLD.

### Morphology and grammatical marking

The findings of the present review suggest that morphology and grammatical marking represent one of the strongest areas of overlap between ASD accompanied by language impairment (ASD-LI) and DLD. Across a range of grammatical domains, including aspect marking, clitic production, article choice, definiteness marking, pronoun interpretation, tense morphology, and broader morphosyntactic abilities, children with ASD-LI frequently showed performance similar to that of children with DLD (Chen et al., 2022; Durrleman and Delage, 2016; Huang and Finestack, 2020; Prévost et al., 2018; Schaeffer et al., 2017; Zhang et al., 2024). In many studies, the two groups did not differ significantly on standardized grammatical assessments. Taken together, these findings are consistent with the notion that morphosyntactic impairment may represent a partially overlapping structural language phenotype across diagnostic categories (Roberts et al., 2004; Whitehouse et al., 2008).

The overlap reported across languages and assessment methods suggests that difficulties in grammatical morphology are not specific to a particular linguistic system but may reflect common underlying vulnerabilities in language acquisition and processing. Similar patterns of impairment were reported in English, French, Mandarin, and Dutch-speaking populations, indicating that grammatical difficulties in ASD-LI and DLD extend across diverse linguistic contexts (Chen et al., 2022; Durrleman and Delage, 2016; Huang and Finestack, 2020; Prévost et al., 2018). Furthermore, several studies demonstrated that morphosyntactic performance was more strongly associated with language impairment status than with diagnostic category, suggesting that structural language abilities may cut across traditional diagnostic boundaries (Ellis Weismer et al., 2017; Huang and Finestack, 2020).

At the same time, the review identified important qualitative differences between the groups. Whereas children with DLD typically exhibited errors reflecting core grammatical weaknesses, children with ASD often demonstrated greater heterogeneity and difficulties influenced by referential processing, discourse integration, perspective-taking, and social-cognitive demands (Durrleman and Delage, 2016; Schaeffer et al., 2017; Zhang et al., 2024). For example, difficulties with first-person clitics, pronoun interpretation, and definiteness marking in ASD were frequently linked to Theory of Mind, discourse processing, or referential challenges rather than to grammatical deficits alone (Durrleman and Delage, 2016; Schaeffer et al., 2017). These findings suggest that similar surface-level performance may arise from partially distinct underlying mechanisms.

Another important finding was the marked heterogeneity of grammatical abilities within ASD. While some children with ASD-LI displayed morphosyntactic profiles that closely resembled those of children with DLD, others demonstrated relatively intact grammatical abilities despite broader communication difficulties (Durrleman and Delage, 2016; Ellis Weismer et al., 2017; Schaeffer, 2018). This variability supports contemporary models proposing multiple language phenotypes within autism and highlights the importance of considering language status, rather than diagnosis alone, when examining structural language abilities.

Overall, the evidence suggests that morphosyntactic impairment represents an important area of convergence between ASD-LI and DLD. However, the findings also indicate that similar grammatical outcomes may reflect different developmental pathways, with DLD characterized by more consistent grammatical deficits and ASD involving additional influences from social-cognitive, discourse, attentional, and working-memory processes (Durrleman and Delage, 2016; Ellis Weismer et al., 2017; Schaeffer et al., 2017; Zhang et al., 2024). Future research should combine standardized language assessments with detailed error analyses and experimental methodologies to better distinguish shared structural language deficits from ASD-specific processing characteristics.

### Syntax and complex sentence processing

Syntax and complex sentence processing emerged as one of the clearest areas of partial overlap between ASD-LI and DLD. Across studies, both groups demonstrated difficulties with syntactically complex structures, including wh-questions, relative clauses, passives, clitic pronouns, reflexives, sentential complements, control structures, and noncanonical word order (Dai et al., 2025; Fortunato-Tavares et al., 2015; Garrido et al., 2024; Moreno-Pérez et al., 2020; Prévost et al., 2017). These findings suggest that children with ASD-LI and DLD share vulnerabilities in processing constructions that place high demands on syntactic computation, movement operations, hierarchical dependency formation, and working memory. The similar performance reported across many syntactic tasks is consistent with proposals that ASD-LI and DLD share vulnerabilities in the processing of complex grammatical structures. In particular, both groups were sensitive to increases in syntactic complexity and frequently showed reduced performance when sentence comprehension required establishing long-distance dependencies, resolving intervention effects, or processing noncanonical word order.

Several studies indicated that this overlap is particularly evident when tasks require comprehension or production of movement-derived structures. Children with ASD-LI and DLD often performed below typically developing peers and showed comparable sensitivity to syntactic complexity, especially in wh-questions, passive constructions, relative clauses, and other noncanonical sentences (Dai et al., 2025; Garrido et al., 2024; Prévost et al., 2017). Similarly, similar performance on reflexive and clitic pronoun comprehension suggests that some aspects of complex syntactic processing may not reliably distinguish ASD from DLD when language level is controlled (Moreno-Pérez et al., 2020). Intervention findings further support this overlap, as children with ASD and DLD showed broadly similar responsiveness to syntactic training targeting expressive and receptive syntax or sentential complement clauses (Azevedo et al., 2025; Durrleman et al., 2017, 2019).

At the same time, the reviewed studies consistently showed that similar accuracy scores may conceal important qualitative differences. Children with DLD tended to exhibit more selective morphosyntactic impairments, with errors largely constrained by grammatical structure and syntactic complexity. In contrast, children with ASD more frequently demonstrated heterogeneous performance profiles, contextually inappropriate responses, perseveration, discourse-related errors, and difficulties linked to referential or pragmatic processing (Nie et al., 2025; Sukenik and Friedmann, 2018; Sukenik et al., 2021). These differences were especially evident in tasks involving wh-question production, ba/bei constructions, and movement-derived dependencies, where overall performance sometimes appeared similar but error types differed substantially.

The findings also suggest that different syntactic constructions may reveal distinct mechanisms across the two disorders. For example, children with SLI/DLD showed particular vulnerability in object relative clauses and other structures involving A′-movement and intervention effects, whereas children with ASD sometimes showed greater difficulty with subject-control constructions or referentially demanding which-questions (Issa et al., 2023; Dai et al., 2025). Such findings indicate that ASD-LI and DLD do not simply represent the same syntactic impairment. Rather, DLD appears to involve more selective and structurally constrained morphosyntactic deficits, while syntactic difficulties in ASD may be more strongly influenced by discourse integration, referential processing, social cognition, and broader task demands.

Overall, the evidence suggests partial overlap between ASD-LI and DLD in complex syntax, particularly in constructions involving movement, noncanonical word order, and high processing load. However, detailed error analyses and task-specific patterns indicate that similar syntactic performance may arise from different underlying linguistic and cognitive mechanisms. These findings highlight the importance of moving beyond accuracy scores and incorporating qualitative error analysis, online processing measures, and discourse-sensitive syntactic tasks when comparing structural language profiles in ASD and DLD.

### Lexical-semantic development

The findings of the present review suggest overlap between ASD and DLD in lexical-semantic abilities. Across studies, children with ASD and DLD frequently demonstrated similar difficulties in vocabulary knowledge, semantic organization, conceptual categorization, word associations, lexical access, and novel word learning (Gladfelter et al., 2019; Haebig et al., 2015; Ramírez-Santana et al., 2019; Ricketts et al., 2015). Both groups often showed reduced lexical depth and weaker semantic organization compared with TD peers, suggesting that limitations in vocabulary development and semantic representation are common features of language impairment across diagnostic categories.

Several studies further demonstrated similarities in lexical learning processes. Children with ASD and DLD were generally able to acquire new vocabulary under structured learning conditions and benefited from external supports such as written input and multimodal visual-verbal presentation (Gladfelter et al., 2019; Ricketts et al., 2015). These findings suggest that the basic mechanisms supporting word learning remain relatively intact in both groups, despite broader language difficulties. The comparable performance observed in lexical decision tasks and semantic categorization measures also suggests overlap in the organization of lexical-semantic knowledge (Haebig et al., 2015; Ramírez-Santana et al., 2019).

However, important differences emerged regarding the mechanisms underlying lexical-semantic processing. Evidence from behavioral and neurocognitive studies suggests that children with DLD experience more pronounced difficulties in lexical acquisition, semantic network organization, and the consolidation of newly learned lexical representations. Children with DLD demonstrated weaker statistical learning abilities, less efficient fast mapping, reduced semantic network activation, and poorer consolidation of lexical-semantic information over time (Haebig et al., 2017; Knowland et al., 2022). In contrast, lexical-semantic processing in ASD appeared more heterogeneous. Many children with ASD, particularly those without co-occurring language impairment, demonstrated lexical learning and semantic processing patterns that were broadly comparable to those of TD peers (Haebig et al., 2017; Knowland et al., 2022). This finding supports broader evidence that structural language difficulties in ASD are not universal but vary considerably according to language phenotype.

The review also identified qualitative differences in semantic processing that may distinguish ASD from DLD. Whereas children with DLD primarily exhibited weaknesses related to lexical acquisition and semantic representation, children with ASD showed greater difficulty integrating contextual and non-literal meanings. This was particularly evident in figurative language processing, where children with ASD demonstrated a stronger literal interpretation bias and reduced activation of idiomatic meanings despite otherwise comparable vocabulary knowledge (Walenski and Love, 2017). Such findings suggest that lexical-semantic difficulties in ASD may be influenced not only by language abilities but also by broader challenges in contextual integration and meaning interpretation.

Taken together, the evidence suggests partial overlap in vocabulary development, semantic organization, and lexical-semantic performance between ASD and DLD. Nevertheless, the underlying nature of these difficulties appears to differ. DLD is more consistently associated with impairments in lexical learning, semantic network formation, and consolidation processes, whereas lexical-semantic performance in ASD is characterized by greater heterogeneity and additional difficulties involving contextual, figurative, and integrative aspects of meaning. These findings are consistent with the view that similar performance on standardized vocabulary and semantic measures may mask distinct cognitive and linguistic mechanisms across the two disorders.

### Narrative and discourse-level language

Narrative findings are consistent with the conclusion that ASD and DLD show partial overlap in structural language, particularly at the level of narrative microstructure. Across studies, both groups demonstrated weaknesses in mean length of utterance, syntactic complexity, lexical diversity, semantic richness, and narrative productivity compared with TD peers (Govindarajan and Paradis, 2022; Nielsen et al., 2025; Norbury et al., 2014). These findings suggest that narrative tasks are sensitive to structural language vulnerabilities in both disorders because they require children to integrate vocabulary, grammar, and sentence-level organization in connected speech.

At the same time, narrative studies revealed clearer differentiation at the macrostructural and discourse-pragmatic levels. Children with ASD more frequently showed difficulties with story coherence, referential cohesion, internal reactions, mental-state language, character introduction, and the organization of socially relevant narrative content (Govindarajan and Paradis, 2022; Norbury et al., 2014). These difficulties appear to reflect the additional social-cognitive and pragmatic demands of narrative production, rather than structural language weakness alone. In contrast, narrative difficulties in DLD appeared more closely tied to underlying linguistic limitations, including reduced syntactic complexity, shorter utterances, and weaker lexical-semantic content (Govindarajan and Paradis, 2022; Norbury et al., 2014).

The findings of Nielsen et al. (2025) further indicate that narrative performance in both ASD and DLD is influenced by demands on information organization and integration. Both groups produced fewer relevant content elements than typically developing peers, suggesting difficulties with macrostructural organization. However, autistic children produced more relevant information when animations were segmented, indicating that memory and discourse-organization demands may particularly affect narrative performance in ASD (Nielsen et al., 2025). This finding supports the interpretation that narrative difficulties in ASD may partly reflect challenges in organizing and integrating information across discourse, even when intentionality attribution itself is relatively intact.

Overall, narrative evidence suggests that microstructural measures primarily capture overlapping structural language characteristics across ASD and DLD, whereas macrostructural and discourse-level measures may be more useful for distinguishing the two disorders. Narrative assessment therefore has particular clinical value because it simultaneously captures structural language skills and broader communicative abilities. Measures such as mean length of utterance, syntactic complexity, and lexical diversity may identify shared language vulnerabilities, while referential cohesion, internal-state language, story coherence, and socially meaningful content may help differentiate ASD from DLD (Govindarajan and Paradis, 2022; Nielsen et al., 2025; Norbury et al., 2014).

### Structural language, cognition, and underlying mechanisms

The findings of the present review suggest that the degree of overlap between ASD and DLD depends partly on the linguistic and cognitive demands of the task. Across many studies, children with ASD-LI and DLD showed similar performance on measures of structural language, including vocabulary, morphosyntax, and complex syntax. However, differences between the groups became increasingly apparent when successful performance required the integration of structural language with discourse context, pragmatics, prosody, Theory of Mind, or other domain-general cognitive processes (Andrés-Roqueta and Katsos, 2020; Eberhardt and Nadig, 2016; Huang et al., 2025).

Several studies demonstrated that structural language ability is a major determinant of performance across both disorders. For example, Andrés-Roqueta and Katsos (2020) found that children with ASD and DLD performed similarly on linguistic-pragmatic tasks that primarily relied on structural language abilities, with structural language emerging as the strongest predictor of performance. Likewise, Eberhardt and Nadig (2016) showed that context-sensitive language comprehension was more strongly associated with language ability than with diagnostic status. Children with ASD and language impairment demonstrated patterns of performance similar to those observed in children with LI, whereas ASD children with intact language abilities performed similarly to TD peers. Together, these findings suggest that some language-related difficulties traditionally associated with ASD may be more closely related to co-occurring language impairment than to autism itself.

At the same time, several studies indicated that superficially similar levels of structural language performance may be supported by different underlying mechanisms. Structural language weaknesses appear to represent an important area of overlap between ASD-LI and DLD, whereas differences become increasingly evident when language processing requires coordination with social-cognitive, pragmatic, discourse-level, or prosodic information. DLD appears to be characterized more consistently by core structural language impairments, while language difficulties in ASD often reflect the interaction between structural language weaknesses and broader communicative, cognitive, and social-processing factors (Andrés-Roqueta and Katsos, 2020; Huang et al., 2025). Consequently, similar performance on standardized language measures should not be interpreted as evidence of identical language phenotypes.

Overall, the reviewed evidence suggests that the distinction between ASD and DLD is most apparent at the interface between structural language and broader communicative processes. While the two disorders frequently show overlapping structural language characteristics, ASD is more consistently associated with difficulties integrating linguistic information with context, prosody, discourse, and social cognition. This may explain why children with ASD and DLD often achieve similar scores on formal language assessments while exhibiting different patterns of language use in naturalistic communication.

### General discussion

The present systematic review synthesized evidence from 44 studies examining structural language abilities in children with Autism Spectrum Disorder (ASD) and Developmental Language Disorder (DLD). Across phonological, morphological, syntactic, lexical-semantic, and narrative domains, the findings suggested a pattern of partial overlap between the two conditions, particularly among children with ASD and co-occurring language impairment (ASD-LI). At the same time, important differences emerged in the severity, consistency, and underlying mechanisms of language difficulties, consistent with the view that ASD-LI and DLD represent partially overlapping but distinct language phenotypes.

One of the most consistent findings across studies was that structural language impairment is not unique to either diagnostic category. Children with ASD-LI frequently demonstrated language profiles similar to those of children with DLD across receptive and expressive language, morphosyntax, vocabulary, and repetition-based measures (Brignell et al., 2018; Georgiou and Spanoudis, 2021; Oi et al., 2017; Ramírez-Santana et al., 2019). Furthermore, repetition-based markers such as nonword repetition and sentence repetition often did not reliably distinguish ASD-LI from DLD, suggesting that these measures may be markers of language impairment more generally rather than disorder-specific indicators (Georgiou and Spanoudis, 2021; Taylor et al., 2014).

The findings also support dimensional approaches to neurodevelopmental disorders. Across several studies, language performance was more strongly associated with language impairment status, working memory, and nonverbal cognitive abilities than with diagnostic category itself (Brignell et al., 2018; Ellis Weismer et al., 2017; Oi et al., 2017). Children with ASD without language impairment often performed similarly to TD peers, whereas children with ASD-LI closely resembled children with DLD. These findings are consistent with theoretical accounts proposing that language impairment represents a dimension that cuts across diagnostic boundaries rather than a characteristic unique to a single disorder (Bishop, 2010a,b; Tager-Flusberg and Joseph, 2003).

Despite this overlap, the review identified several characteristics that differentiate ASD from DLD. Across studies, structural language impairments tended to be more severe and pervasive in DLD, particularly in phonology, morphosyntax, vocabulary, and literacy-related skills (Andreou et al., 2025; Dockrell et al., 2019; Doghadze and Gagoshidze, 2023). In contrast, language abilities in ASD were characterized by greater heterogeneity. Some ASD children demonstrated language profiles similar to those observed in DLD, whereas others showed relatively intact structural language despite persistent social-communication difficulties (Gibson et al., 2013; Hare-Harris et al., 2019). This variability supports contemporary models proposing multiple language phenotypes within the autism spectrum (Silleresi, 2023; Vogindroukas et al., 2022).

Importantly, the distinction between ASD and DLD became increasingly evident when language tasks extended beyond core structural language and required integration with discourse, pragmatics, prosody, or social cognition. Children with ASD frequently demonstrated difficulties in referential processing, narrative coherence, mental-state language, figurative language comprehension, discourse integration, and context-sensitive interpretation, even when structural language performance was similar to that of children with DLD (Andrés-Roqueta and Katsos, 2020; Govindarajan and Paradis, 2022; Walenski and Love, 2017). These findings suggest that structural language difficulties in ASD often interact with broader social-cognitive and communicative processes, whereas language difficulties in DLD are more consistently associated with core linguistic mechanisms.

The considerable heterogeneity observed among children with ASD and co-occurring language impairment also has important clinical implications for differential diagnosis. The present review suggests that structural language measures alone may be insufficient to distinguish some children with ASD-LI from children with DLD, particularly during early childhood when language profiles are still emerging. Consequently, assessment should extend beyond standardized structural language testing and incorporate developmental history, social communication, pragmatic language abilities, adaptive functioning, cognitive profile, and behavioral observations. A multidisciplinary approach involving speech-language pathologists, psychologists, developmental pediatricians, and other relevant professionals may facilitate more accurate differentiation between children with primary DLD and those whose structural language difficulties occur within the broader neurodevelopmental profile of ASD. Longitudinal follow-up may be particularly valuable for late-talking children or those presenting with atypical structural language profiles, as developmental trajectories often provide important diagnostic information that may not be apparent during a single assessment.

The findings further indicate that similar levels of behavioral performance may mask distinct underlying cognitive and linguistic processes. Several studies demonstrated that children with ASD and DLD achieved comparable scores on grammatical, syntactic, and lexical tasks while exhibiting different error patterns and processing strategies (Durrleman and Delage, 2016; Nie et al., 2025; Sukenik and Friedmann, 2018). Children with DLD generally produced errors reflecting grammatical and linguistic limitations, whereas children with ASD more frequently demonstrated pragmatic, discourse-related, or contextually inappropriate responses. Consequently, reliance solely on standardized language scores may underestimate important qualitative differences between the two disorders. Qualitative analysis of error patterns may therefore complement standardized language assessments by providing additional insight into the underlying linguistic and cognitive mechanisms contributing to language difficulties, thereby supporting more accurate differential diagnosis and intervention planning.

Taken together, the evidence is consistent with a model of partial phenotypic overlap. Structural language impairment appears to represent an important area of overlap between ASD-LI and DLD, particularly in morphosyntax, complex syntax, vocabulary, and phonological processing. However, the greater heterogeneity of ASD, the more severe structural impairments typically observed in DLD, and the distinctive social-pragmatic challenges associated with ASD suggest that the two conditions cannot be considered equivalent manifestations of a single language disorder. Importantly, although ASD-LI and DLD share several structural language characteristics, qualitative differences in error patterns and processing strategies suggest that similar behavioral performance on standardized language measures may arise from distinct underlying linguistic and cognitive mechanisms. Rather, the two conditions appear to share certain developmental pathways while also reflecting disorder-specific mechanisms that shape language development in different ways.

However, these conclusions should be interpreted with caution. Many of the included studies were based on relatively small samples, and the absence of statistically significant differences between ASD-LI and DLD should not be interpreted as evidence of equivalence. Rather, the available evidence suggests overlapping performance across several structural language measures, while acknowledging that some studies may have been underpowered to detect modest between-group differences.

## Limitations of the available evidence

Several limitations of the available evidence should be considered when interpreting the findings of this review. First, considerable heterogeneity existed across studies in participant characteristics, diagnostic criteria, age ranges, language abilities, and assessment methods. Many studies included mixed ASD samples, whereas others specifically examined ASD with language impairment (ASD-LI), making direct comparisons across studies challenging.

Second, the reviewed studies employed a wide variety of language measures, including standardized assessments, experimental tasks, language samples, neurocognitive measures, and narrative analyses. Although this diversity provided a comprehensive picture of language functioning, it also limited direct comparisons across studies and prevented quantitative synthesis.

Third, most included studies used cross-sectional designs. Consequently, relatively little is known about the developmental trajectories of structural language abilities in ASD and DLD or about how similarities and differences evolve across childhood and adolescence. Longitudinal research is needed to determine whether overlapping language characteristics reflect common developmental mechanisms or merely similar behavioral outcomes at specific developmental stages.

Fourth, many studies involved relatively small sample sizes, which may limit statistical power and the generalizability of findings. Accordingly, the absence of statistically significant differences between ASD-LI and DLD in individual studies should not be interpreted as evidence that the groups are equivalent, as some studies may have been underpowered to detect modest between-group differences. In addition, relatively few studies incorporated neurocognitive, psycholinguistic, or online-processing methodologies. As suggested by several reviewed studies, similar behavioral performance may conceal important differences in underlying processing mechanisms. Future research should integrate behavioral, neurocognitive, and neurobiological approaches to better understand the sources of overlap and divergence between ASD and DLD.

Fifth, the available literature gave limited consideration to sex differences. Although participant sex was reported in many studies and ASD samples were predominantly male, few studies examined sex as an independent variable or reported sex-specific analyses of structural language outcomes. Consequently, the current evidence does not permit conclusions regarding the influence of sex on the structural language similarities and differences observed between ASD and DLD. Future research should routinely examine sex-specific language profiles to determine whether structural language phenotypes differ between males and females.

## Limitations of the review

Several limitations of the review process should also be acknowledged. Only studies meeting the predefined inclusion criteria were considered, and relevant unpublished studies may have been missed. Due to substantial clinical and methodological heterogeneity across the included studies, together with considerable variation in participant characteristics, diagnostic classifications, assessment instruments, outcome measures, and statistical reporting, a quantitative meta-analysis was not conducted and the findings were synthesized narratively. Furthermore, many studies were based on relatively small samples and did not report sufficient statistical information to permit the calculation of comparable effect sizes across language domains. Consequently, the review should not be interpreted as demonstrating equivalence between ASD-LI and DLD based solely on the absence of statistically significant differences reported in individual studies. Rather, the conclusions reflect the overall pattern of evidence across the available literature and should be interpreted with appropriate caution. Future quantitative syntheses focusing on more homogeneous language domains and standardized outcome measures may provide more precise estimates of similarities and differences between ASD-LI and DLD.

## Future directions

Future studies should examine language phenotypes within ASD more systematically, distinguishing children with ASD-LI from those with typical language abilities. Greater attention should also be given to cross-linguistic research, as much of the available evidence originates from a limited number of languages. Finally, future investigations should focus on identifying markers that reliably differentiate ASD-LI from DLD while simultaneously exploring shared mechanisms that may inform transdiagnostic models of language impairment.

## Clinical implications

The findings of this review have important implications for assessment, diagnosis, and intervention. First, the pattern of overlap observed between ASD-LI and DLD suggests that clinicians should avoid assuming that language difficulties are disorder-specific. Comprehensive language assessment should evaluate structural language domains, including phonology, morphology, syntax, semantics, and discourse, regardless of diagnostic category.

Second, the findings highlight the limitations of relying exclusively on standardized language measures for differential diagnosis. Because children with ASD-LI and DLD frequently obtain similar scores on formal language assessments, clinicians should incorporate detailed language-sample analysis, narrative assessment, error analysis, and pragmatic evaluation into diagnostic protocols. Measures of narrative macrostructure, referential cohesion, mental-state language, discourse organization, and pragmatic competence may provide valuable information for distinguishing ASD from DLD.

Third, the evidence suggests that intervention approaches targeting structural language may benefit children across both disorders. Evidence of similar responsiveness to morphosyntactic and syntactic interventions suggests that many evidence-based language interventions developed for DLD may also be effective for children with ASD-LI. However, intervention for ASD should additionally address discourse, pragmatic language, social communication, perspective-taking, and contextual language use.

Finally, the findings support individualized assessment and intervention planning. Given the substantial heterogeneity observed within ASD, language status should be considered separately from diagnostic category when making clinical decisions. Identifying ASD-LI as a distinct subgroup may improve treatment planning, prognostic evaluation, and educational support.

## Conclusion

This systematic review suggests that children with ASD-LI and DLD share substantial structural language difficulties across phonological, morphological, syntactic, lexical-semantic, and narrative domains. Similarities were particularly evident in morphosyntax, complex syntax, vocabulary, and repetition-based measures, consistent with partially overlapping language phenotypes across the two disorders. However, important differences were also identified. Structural language impairments tended to be more severe and consistent in DLD, whereas ASD was characterized by greater heterogeneity and additional difficulties involving discourse, pragmatics, contextual integration, and social cognition.

Overall, the findings are consistent with a model of partial phenotypic overlap rather than diagnostic equivalence. Structural language impairment appears to represent an important area of overlap between ASD-LI and DLD, but the underlying mechanisms and broader communicative profiles differ across disorders. These findings emphasize the importance of moving beyond diagnostic labels and considering language abilities dimensionally when assessing, researching, and supporting children with neurodevelopmental disorders.

## Data Availability

The original contributions presented in the study are included in the article/Supplementary material, further inquiries can be directed to the corresponding author.
