# Supplementary material for: Structural language profiles in children with autism spectrum disorder and developmental language disorder: a systematic review
Source: Front Psychol. 2026 Sep 3;17:1920573. doi: 10.3389/fpsyg.2026.1920573 (PMC13582585; doi:10.3389/fpsyg.2026.1920573)
Supplement: Supplementary file 1 [file Data_Sheet_1.PDF]

Supplementary Table S1: Database-specific search strategies

Database/platform	Date last searched	Search strategy	Limits applied
PubMed (NCBI)	12 November 2025	("Autism Spectrum Disorder"[Mesh] OR "autism"[Title/Abstract] OR ASD[Title/Abstract] OR "autism spectrum disorder"[Title/Abstract] OR "autistic disorder"[Title/Abstract] OR "high functioning autism"[Title/Abstract] OR HFA[Title/Abstract] OR Asperger*[Title/Abstract]) AND ("Language Disorders"[Mesh] OR "developmental language disorder"[Title/Abstract] OR DLD[Title/Abstract] OR "specific language impairment"[Title/Abstract] OR SLI[Title/Abstract] OR "language impair*"[Title/Abstract] OR "language disorder*"[Title/Abstract] OR "language delay*"[Title/Abstract]) AND ("structural language"[Title/Abstract] OR "language phenotype"[Title/Abstract] OR "language profile"[Title/Abstract] OR phonolog*[Title/Abstract] OR morpholog*[Title/Abstract] OR syntax[Title/Abstract] OR semantics[Title/Abstract] OR grammar[Title/Abstract]) AND ("Child"[Mesh] OR "Child, Preschool"[Mesh] OR "Adolescent"[Mesh] OR child*[Title/Abstract] OR preschool*[Title/Abstract] OR toddler*[Title/Abstract] OR	English language; publication date 1 January 2013–12 November 2025

		pediatric[Title/Abstract] OR paediatric[Title/Abstract] OR "school-age"[Title/Abstract] OR adolescen*[Title/Abstract])	
APA PsycInfo (via PsycNet)	12 November 2025	("autism" OR ASD OR "autism spectrum disorder" OR "autistic disorder" OR "high functioning autism" OR HFA OR Asperger*)<br>AND<br>("developmental language disorder" OR DLD OR "specific language impairment" OR SLI OR "language impair*" OR "language disorder*" OR "language delay*")<br>AND<br>("structural language" OR "language phenotype" OR "language profile" OR phonolog* OR morpholog* OR syntax OR semantics OR grammar)<br>AND<br>(child* OR preschool* OR toddler* OR pediatric OR paediatric OR "school-age" OR adolescen*)	English language; 2013–2025; journal articles
ERIC	12 November 2025	("autism" OR ASD OR "autism spectrum disorder" OR "autistic disorder" OR "high functioning autism" OR HFA OR Asperger*) AND ("developmental language disorder" OR DLD OR "specific language impairment" OR SLI OR "language impair*" OR "language disorder*" OR "language delay*") AND ("structural language" OR "language phenotype" OR "language profile" OR phonolog* OR morpholog* OR syntax OR semantics OR grammar) AND (child* OR	English language; 2013–2025; journal articles

		preschool* OR toddler* OR pediatric OR paediatric OR "school-age" OR adolescen*)	
Google Scholar	12 November 2025	Supplementary searches were conducted using combinations of the core concepts (autism, developmental language disorder, structural language) together with citation searching of eligible studies.	Used only for supplementary searching and citation tracking
Semantic Scholar	12 November 2025	Supplementary searches were conducted using combinations of the core concepts and citation searching to identify additional relevant studies.	Used only for supplementary searching and citation tracking
Reference-list screening	12 November 2025	Reference lists of included studies were manually examined for additional eligible records	No additional database limits
Note. PubMed (NCBI), ERIC, and APA PsycInfo (via PsycNet) were treated as the primary bibliographic databases. Search syntax was adapted to the indexing system and search functionality of each database while maintaining the same conceptual framework. Google Scholar and Semantic Scholar were used only as supplementary sources for broader literature retrieval and citation searching because searches conducted through these platforms are less reproducible than searches in bibliographic databases.			
